# Genetically engineered electrospinning contributes to spinal cord injury repair by regulating the immune microenvironment

**DOI:** 10.3389/fbioe.2024.1415527

**Published:** 2024-06-12

**Authors:** Yang Sun, Jie Wu, Liang Zhou, Wei Wang, Haibo Wang, Shaosong Sun, Yichang Xu, Lichen Zhang, Xinzhao Jiang, Guoqing Zhu, Kun Xi, Yong Gu, Liang Chen

**Affiliations:** ^1^ Department of Orthopedics, The First Affiliated Hospital of Soochow University, Suzhou, Jiangsu, China; ^2^ Institute Orthopedic, Soochow University, Suzhou, Jiangsu, China; ^3^ Department of Orthopedics, Suzhou Municipal Hospital, Suzhou, Jiangsu, China

**Keywords:** genetically engineered, immunoregulation, tissue engineering, spinal cord injury, nerve repair

## Abstract

**Introduction:** Spinal cord injury (SCI) is associated with microenvironment imbalance, thereby resulting in poor regeneration and recovery of the spinal cord. Gene therapy can be used to balance the inflammatory response, however target genes cannot exist in localized injured areas.

**Methods:** A genetically engineered electrospun scaffold (GEES) to achieve long-term immunoregulation and nerve repair was constructed. By combining the microfluidic and electrospinning techniques, interleukin-10 plasmid (pIL10) was loaded into lipid nanoparticles (LNPs) (pIL10-LNP), which was encapsulated to the nerve growth factor (NGF). Immunofluorescence staining, qRT-PCR, ELISA, flow cytometry, and other tests were employed to comprehensively assess the role of GEES in modulating macrophage polarization and facilitating neural repair.

**Results:** The results showed that the scaffold released >70% of the pIL10-LNP within 10 d and continued slow release within 30 d. *In vitro* cell experiments have demonstrated that GEES effectively stimulates macrophages to secrete anti-inflammatory cytokines and facilitates the differentiation of neural stem cells into neuronal cells. In rat T9 SCI model, the GEES significantly inhibited the inflammatory response in the acute and chronic phases of SCI by transfecting local tissues with slow-release pIL10-LNP to promote the release of the anti-inflammatory factor IL10, thereby creating a favorable microenvironment. With the addition of NGF, the repair and regeneration of nerve tissues was effectively promoted, and the post-SCI motor function of rats improved.

**Discussion:** GEES can regulate post-SCI immune responses through continuous and effective gene delivery, providing a new strategy for the construction of electrospun scaffolds for nerve repair in gene therapy.

## 1 Introduction

Spinal cord injury (SCI) is an intractable disease caused by trauma that results in sensory and motor dysfunction below damaged spinal segments, thereby causing permanent impairment, for which there are no effective treatments (Courtine and Sofroniew, 2019; Woo et al., 2004). Globally, there are approximately 180,000 new cases of SCI each year, imposing a huge burden on patients, their families, and the society (Bradbury and Burnside, 2019). SCI is often classified as a primary or secondary injury. Whereby, primary injury refers to localized spinal cord compression, often caused by trauma, which is irreversible due to limitations in nerve regeneration (Fakhoury, 2015) and secondary injury, which is the acute inflammatory response after SCI. SCI is an important pathogenesis, and both microglial activation and peripheral macrophage infiltration in the spinal cord often reach a peak within 7 days after injury (Wynn and Vannelle, 2016; Tran et al., 2018; Hellenbrand et al., 2021; Hoang et al., 2022). In addition, pro-inflammatory factors (TNF-α, etc.) not only promote the activation of immune cells into M1 macrophages that worsen the local inflammatory response, but also enhance the aggregation of reactive astrocytes into dense glial scars to fill in the post-SCI cavity, which become an obstacle to nerve and axon regeneration, seriously affecting the prognosis of SCI (Liu et al., 2019; Park et al., 2019; Venkatesh et al., 2019; Sterner and Sterner, 2023; Tian et al., 2023). Therefore, regulating the inflammatory response is an important strategy for SCI treatment. In recent years, bioengineered tissue implantation has gradually become a new approach for SCI treatment. Implanted bioengineered scaffolds can bridge the injury site, but effectively balancing the polarization of M1 and M2 macrophages in the inflammatory response during the acute phase of SCI after implantation remains an urgent problem (Cui et al., 2010; Li et al., 2015; Angel et al., 2022). Therefore, it is necessary to construct a platform to precisely regulate the polarization of immune cells in the early stage of SCI to alleviate inflammatory responses and synergistically promote the differentiation and repair of neural stem cells (NSCs).

Interleukin-10 (IL-10) is a pleiotropic cytokine with potent anti-inflammatory properties in the chemokine family and is mainly secreted by activated T cells, monocytes, B cells, and macrophages; it weakens antigen presentation by downregulating the expression of major histocompatibility complex II (MHC II) on the surface of macrophages, decreases the activity of T lymphocytes, and inhibits activation, migration, and adhesion of inflammatory cells. Meanwhile, it can attenuate the immuno-inflammatory response by activating anti-inflammatory cytokines such as TNF-α, IL-6, and IL-1 in macrophages (Saraiva and OGara, 2010; Glocker et al., 2011; Mannino et al., 2015; Hedrich and Bream, 2010; Devkota et al., 2012). Additionally, it is important to promote the repair and regeneration of nerve tissues and immunoregulation after injury. Nerve growth factor (NGF) is involved in the regulation of nerve cell growth, development, differentiation, and post-injury repair of the nervous system. It can drive the expression of genes, such as *Bcl-2*, by binding to TrkA receptors, thereby stimulating the proliferation and survival of target neurons and exerting an important regulatory effect on the functional expression in the central nervous system (CNS). However, in acquired immunity, NGF is often produced by CD4^+^ T cells and released at high concentrations by mastocytes during inflammation to induce the axonal growth of nociceptive neurons (Bannwarth and Kostine, 2014; Chen et al., 2017; Minnone et al., 2017; Hosein et al., 2022; Maple-Grødem et al., 2023), which is contrary to the expected immune suppression after SCI. Therefore, tissue-engineered scaffolds used in SCI repair should continuously release NGF to promote neuronal and axonal growth, while suppressing the inflammatory response.

Biomaterial-based regenerative medicine shows good prospects for SCI repair. Our research group previously developed oriented microsol electrospun fibers mimicking the neural extracellular matrix, characterized by a simple process, stable properties, drug loading rate of up to 80%, slow release ability within 6 weeks, and successfully combined cationic liposomes with electrospun scaffolds through acid-sensitive Schiff base bonds. Thus, a programmed gene transfection and biological factor nanofiber delivery system was established, endowing electrospinning with the capacity of non-viral gene transfection and excellent immunoregulation (Zhou et al., 2014; Xi et al., 2021). The core-shell structure of microsol electrospinning can protect biological factors from the local inflammatory environment of SCI (Zhang et al., 2019), and its internal structure provides space for secondary modification of nanoparticles. Lipid nanoparticles (LNPs) are capable of effectively encapsulating, protecting, and delivering mRNA and pDNA into cells. Additionally, they have been used as mature drug delivery systems with a high rate of transfection with cationic liposomes, low cytotoxicity, and low immunogenicity (Lonez et al., 2012; Pugliese et al., 2018). More importantly, the nanoscale particle size of LNPs make it possible to enter the “core” of the microsol electrospun filaments, therefore a fixed number of preparations can be accurately encapsulated to improve the dose accuracy. Based on the properties of localized drug delivery, tissue-engineered electrospun scaffolds carrying gene vectors can release and deliver therapeutic genes to locally targeted implantation sites, which is an ideal and efficient method of delivering target genes. The double-layer “shell” structure of the electrospun filament-encapsulated nanoparticles provides double protection for gene vectors and allows for the efficient transfection of local cells and tissues, as well as improving the unity of materials.

**FIGURE 1:**
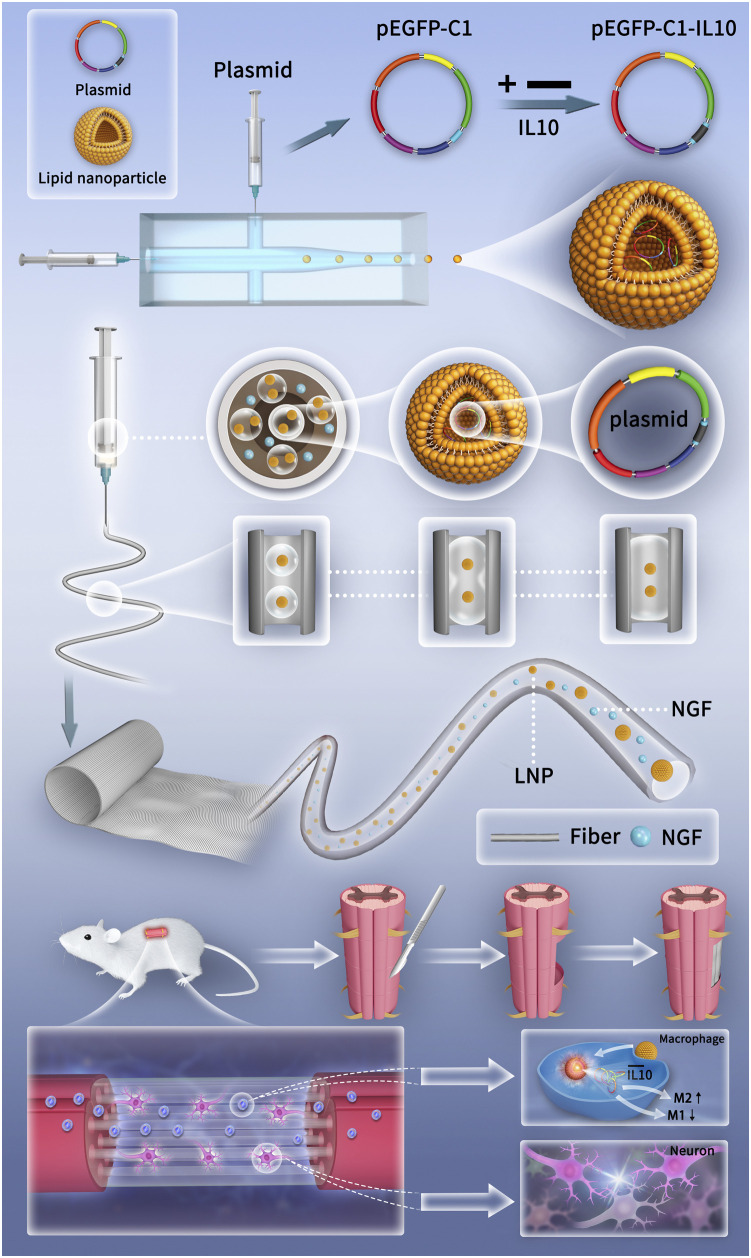
Schematic diagrams of the preparation of genetically engineered electrospun scaffolds and the mechanism of promoting SCI repair.

In this study, microfluidic and electrospinning techniques were combined to design a genetically engineered electrospun scaffold (GEES) with the goal of regulating the balance between inflammatory response and nerve repair after SCI. First, recombinant IL-10 plasmids (pIL10) were transformed into LNPs (pIL10-LNP) using microfluidic chips. The LNPs were then homogeneously dispersed in a hyaluronic acid (HA) solution using microsol electrospinning, and the outer layer was wrapped with poly L-lactic acid (PLA) to protect the target gene (Figure 1). The results showed that pIL10-LNP could be effectively transfected into macrophages in the SCI area to contribute to M2-type polarization of macrophages and secretion of anti-inflammatory factors and stabilize the pro-inflammatory/anti-inflammatory balance in the acute phase of SCI, thus alleviating the dramatic inflammatory response after SCI and creating a good microenvironment for subsequent nerve regeneration. This study systematically assessed and verified that GEES could safely and effectively inhibit the inflammatory response and promote nerve tissue repair after SCI in three ways: 1) characterization of the biological and physical properties of the electrospun scaffold; 2) evaluation of the electrospun scaffold for immunoregulation and pro-nerve growth effects of macrophages and NSCs *in vitro*; and 3) evaluation of the electrospun scaffold for immunoregulation, nerve regeneration, and motor functional recovery in rats *in vivo*. These findings provide a new option for gene-biological tissue-engineered scaffold treatment of SCI.

## 2 Materials and methods

Preparation and characterization of pIL10-LNP:

Preparation: A liposome solution was prepared using 78.1 mg of lecithin (Yuanye, Shanghai, China), 16.7 mg of cholesterol (Arcos, Belgium) and 2 mg of octadecylamine (Aladdin, Shanghai, China) dissolved in 3 mL of anhydrous ethanol. A 1 mL syringe was used to extract the liposome solution and plasmid solution, where the liposome solution was connected to the middle channel of the microfluidic system (Longer, China), and the plasmid solution was connected to the channels on both sides. The syringe was fixed on a micro syringe pump, with a flow rate of 1 mL/h^−1^ (the middle channel) and 10 mL/h^−1^ (the channels on both sides). The liposomes collected were ultrasonically dispersed homogeneously.

Characterization: TEM (FEI, United States): 2 μL of LNP was dropped on a copper grid and dried at room temperature, and its morphology was observed under a voltage of 120 kV.

Particle size and zeta potential: 2 mL of liposome solution was detected using a dynamic light scattering particle size analyzer (Malvern, United Kingdom) to assess the physical properties of nanoparticles.

Encapsulation efficiency (EE%): 1 mL of pIL10-LNP samples was centrifuged for 15 min (5,000 rpm) and added with 500 μL of deionized water. The operation was repeated three times. Then the liquid at the bottom was collected and quantified according to the instructions of dsDNA Kit (Invitrogen, United States):

$$\text{EE}\%=\frac{\mathrm{Initial}\;\mathrm{pDNA}\;-\;\mathrm{unencapsulated}\;\mathrm{pDNA}}{\text{Initial}\;\text{pDNA}}\times 100\%$$

Transfection efficiency and screening of pIL10-LNP under different ratios: Rat bone marrow macrophages (BMMs) (Orthopaedic Institute, Soochow University) were inoculated in a 24-well plate at 4.0×10^4^ cells/well, and added with 100 μL of pIL10-LNP solution at different ratios (1:1, 1:1.5, 1:2, 1:2.5, and 1:3). 72 h later, the cell nuclei were labeled with DAPI, eGFP images were captured under a fluorescence microscope (Olympus), and the fluorescence co-localization rate was analyzed by ImageJ software. Cell culture supernatants were harvested at 48 h, and the IL-10 secretion amount in BMMs after transfection was detected according to the instructions of the IL-10 ELISA Kit (Abcam, United States). Finally, the optimal pIL10-LNP ratio was screened.

Preparation and characterization of electrospun fibers:

Preparation: 0.02 g of Span-80 (Sigma, United States) was dissolved in 8 g of DCM (Qiangsheng, Jiangsu, China). The prepared LNPs were dissolved in 1% HA (Yuancheng, Wuhan, China), and 200 μL of the mixture was taken, added to DCM and ultrasonically dispersed homogeneously. Then 0.5 g of PLA was added to 4 g of DCM and stirred at room temperature with a magnetic stirrer (Sile, Shanghai, China) until a homogeneous and stable solution was obtained. Then the resulting solution was added with 2 g of dimethylformamide (DMF) (Qiangshun, Shanghai, China) and stirred continuously until a homogeneous transparent electrospun solution was obtained. The resulting electrospun solution was extracted using a 10 mL syringe, and the oriented microsol electrospun fibers were collected using the electrospinning equipment (Truelab, Shanghai, China) under the following parameters: receiving distance: 15 cm, voltage: about 15 kV, propel pump speed: 5 mL/h^−1^, nozzle length: 10 cm, and inner diameter: 0.9 mm. Finally, the electrospun fibers collected were placed in a vacuum desiccator (CHRIST, Germany) overnight to completely remove residual reagents on the surface, and stored at 4°C.

Characterization: Microsol particle size: 2 mL of HA microsol solution was detected for the particle size distribution using the dynamic light scattering particle size analyzer (Malvern, United Kingdom).

SEM: Electrospun fiber scaffolds in PLA, PLA-HA, and pIL10-LNP@PLA-HA groups were fixed on the sample stage, and sprayed with gold on the surface to prevent electrostatic interference. The morphology of scaffolds in each group was observed under a microscope voltage of 10 kV (FEI, United States). 100 fibers were randomly selected in each group and their diameters were quantitatively analyzed using ImageJ software.

TEM: The scaffolds in pIL10-LNP@PLAHA group were randomly sampled using copper mesh. The internal structure of scaffolds were observed under a microscope voltage of 120 kV (FEI, United States).

Detection of WCA: WCA of scaffolds in PLA, PLA-HA, and pIL10-LNP@PLA-HA groups was detected using a water contact angle analyzer (Dataphysics, Germany).

Mechanical properties: The scaffolds in PLA, PLA-HA, and pIL10-LNP@PLA-HAgroups were sampled (length × width × thickness: 15.0 mm × 3.0 mm × 0.1 mm) and subjected to mechanical testing (parameters: mechanical sensor 50 N, tensile speed: 10 mm/min) (Hengyi, Shanghai, China).

Degradation rate: The scaffolds in PLA, PLA-HA, and pIL10-LNP@PLA-HA groups were placed in centrifuge tubes and added with 30 mL of PBS, and PBS was replaced every day. At 0, 5, 10, 15, and 20 weeks, the scaffolds were taken out and freeze-dried to remove the water, and weighed. Finally, the ratio of the weight of the residual scaffolds to that of the initial scaffolds was calculated.

Release rate: The scaffolds in pIL10-LNP@PLA-HA group were immersed in centrifuge tubes containing 10 mL of PBS, and placed in a thermostatic shaker at 120 cycles/min and 37°C. The liquid in the tubes was collected at 0, 1, 3, 5, 10, 15, 20, 25 and 30 days, and added with 10 mL of new PBS. Finally, the release curves of NGF and pIL10-LNP were analyzed.

*In vitro* study—Preparation before cell experiments: Rat NSCs (Orthopaedic Institute, The First Affiliated Hospital of Soochow University) and BMMs (Orthopaedic Institute, The First Affiliated Hospital of Soochow University) were inoculated in the scaffolds of each group at a density of 5 × 10^5^ and 5 × 10^3^ cells/well, respectively, and the medium was replaced every 2 days. They were cultured in a 5% CO_2_ incubator at 37°C and 95% relative humidity, with PLA, PLA-HA, and LNP@PLA-HA as negative controls.

*In vitro* study—Viability and proliferation of BMMs: After co-culture with scaffolds for 24 h in each group, the viability of BMMs was calculated by calcein-AM/propidium iodide (PI) staining (Solarbio, Beijing, China) at room temperature. 15 min later, images were acquired under an inverted fluorescence microscope (Olympus). According to the instructions of CCK-8 reagent (Dojindo), the solution was added into a 96-well plate at 1:10, and 100 μL of the supernatant was transferred into another 96-well plate at one, three, and 7 days, respectively. Then the optical density was measured at 450 nm using a microplate reader (Thermo) to detect cell proliferation status, with blank wells as blank controls, and PLA, PLA-HA and LNP@PLA-HA as negative controls.

*In vitro* study—IF analysis: After co-culture with scaffolds in each group, the BMMs were fixed in 4% paraformaldehyde for 30 min at each time point. Then they were incubated with PBS containing 0.2% Triton X-100 for 1 h at 37°C, and with PBS containing 6% BSA overnight. The next day, they were incubated again with primary antibodies against Arg-1 (Cell Signaling, 93668S, 1:50), iNOS (Affinity, AF0199, 1:200), Tuj-1 (Abcam, ab179513, 1:1,000), NF200 (Abcam, ab82259, 1:200), Tau (Abcam, ab80579, 1:250), NSE (Abcam, ab53025, 1:200), integrin β1 (Proteintech, 26918-1-AP, 1:200), and secondary antibodies (Abcam, ab6939, 1:10,000). Iba-1 (Servicebio, GB12105, 1:1,000) at 4°C overnight, and then with secondary antibodies (Abcam, ab97035, 1:10,000) at 37°C for 1 h. Then cell nuclei were stained with DAPI or Hoechst (Beyotime). The samples were washed three times with PBS between every two steps. Images were captured under the inverted fluorescence microscope (Olympus) and analyzed using ImageJ software.

*In vitro* study—immune regulation: BMMs were seeded in culture plates at a density of 5×10^3^ cells and co-cultured with various materials. Following 3 and 5 days of incubation, culture supernatants were collected post-centrifugation, and the concentrations of IL-10 and TNF-α factors in the supernatant were determined using ELISA kits (Elabscience). The collected cells were co-cultured, and qRT-PCR was employed to detect the expression of IL-10 and TNF-α genes in various BMM groups. The reagents utilized included a reverse transcription kit (MCE) and SYBR RealMasterMix (MCE), while primer sequences were designed by Shanghai Bioengineering (Supplementary Table S1).

*In vitro* study—SEM: After co-culture with scaffolds in each group for 3 days, the medium was aspirated and the BMMs were washed with PBS, fixed in 4% paraformaldehyde for 30 min and washed with PBS, followed by complete dehydration with different concentrations of ethanol solutions (10%, 15%, 20%, 30%, 40%, 50%, 80%, 90%, and 100%). After drying, gold spraying was performed, and the cell morphology on the scaffolds in each group was observed by SEM (10 kV).

*In vivo* study—Animal modeling: The animal study protocol was approved by the Ethics Committee of Soochow University (ID: SUDA20230913A02). Female SD rats (approximately 220 g) were obtained from the Laboratory Animal Center of Soochow University. The rats were anesthetized by intraperitoneal injection of 2% pentobarbital (50 mg/kg), and their back was prepared and sterilized. Then a 3 cm-long incision was made longitudinally with the T9 vertebrae as the center, and the fascial muscles were separated layer by layer. The T9 vertebral plate was removed using rongeur forceps to fully expose the spinal cord. Under the observation of a surgical microscope (ZTGX, China), we delicately clamped and incised the dura mater using forceps, thereby exposing the T9 segment of the spinal cord. Following meticulous measurement, we employed microscissors to execute a right-sided hemisection of the spinal cord at both ends. Subsequently, approximately 3 mm of spinal cord tissue was excised using a surgical knife, followed by compression for hemostasis. The scaffold was rolled into a column and placed at the defect site, and then the incision was sutured layer by layer. Postoperatively, each rat was given 2 × 10^5^ U antibiotics intramuscularly every day for 5 days. The bladder of rats was manually emptied every 12 h postoperatively until they resumed voluntary urination. Laminectomy was performed in sham group, and T9 hemi-transverse spinal cord defect was created without implantation in blank control group. PLA, PLA-HA, and LNP@PLA-HA were used as negative controls.

*In vivo* study—Motor function: The motor functional recovery was assessed by two uninformed researchers using BBB (0-21 points) and IPT scores at fixed time points every week postoperatively. In IPT (Rivlin method), the rats were placed on an inclined plate with a raise from 0° to 5° within 5 s, and the maximal angle of stay on the inclined plate was recorded.

*In vivo* study—Histological analysis: At 1, 4 and 8 weeks postoperatively, the rats were euthanized and perfused with normal saline and 4% paraformaldehyde, and the spinal cord samples were harvested. According to the ethical standards governing animal experimentation, we employed a small animal euthanasia system (CL-1000, YuYanBio) to administer CO_2_ euthanasia to each group of rats. The rats were placed in an euthanasia chamber and exposed to CO_2_ gas (concentration 95%–100%, 10 min) to facilitate rapid induction of death. First, the samples were sectioned and stained with H&E, and the images were acquired using an inverted microscope (Olympus) and analyzed using ImageJ software. For immunohistochemical staining, antigens and non-specific antigens were processed with 0.3% hydrogen peroxide and 5% bovine serum protein, respectively. Then the sections were incubated with primary antibodies against GFAP (Servicebio, GB12096, 1:1,000), Tuj-1 (Abcam, ab179513, 1:1,000), NG2 (Affinity, DF12589, 1:200), GAP-43 (Abcam, ab75810, 1:200), secondary antibodies (Abcam, ab150113, 1:10,000). IL-10 (Proteintech, 60269, 1:200), TNF-α (Proteintech, 60291, 1:200), Iba-1 (Servicebio, GB12105, 1:1,000), iNOS (Proteintech, 18985, 1:200) and Arg-1 (Proteintech, 16001, 1:200) at 4°C overnight, and with secondary antibodies (Abcam, ab97035, 1:10,000) at room temperature for 1 h. Finally, the sections were observed under a fluorescence microscope (Olympus) and analyzed using ImageJ software.

*In vivo* study—Flow cytometry: The rats were euthanized 7 days postoperatively. Spinal cord tissues were harvested from a 5-mm area centered on the injury site and minced, and the cells were mechanically separated. Then the suspension in each group was centrifuged for 10 min (300×g, 4°C), incubated with primary antibodies against CD11b (Biolegend, 201807, 1:200), CD86 (Biolegend, 200305, 1:200) and CD206 (Proteintech, 18704-1-AP, 1:200) and with secondary antibodies (Abcam, ab6717, 1:2000) for 30 min at 4°C, and fixed with paraformaldehyde. Finally, flow cytometry was performed using a flow cytometer (BD FACSAria III), and the results were analyzed using FlowJo 7.6 software.

Statistical analysis: Characterization and *in vitro* and *in vivo* experiments were repeated independently at least three times in each group. All data were described by mean ± standard deviation, and compared among groups by one-way ANOVA and Tukey’s test. Prism 9.0 and Origin 2021 were used for statistical analysis. *p* < 0.05 was considered statistically significant.

## 3 Results

### 3.1 Characterization of pIL10-LNP

**FIGURE 2:**
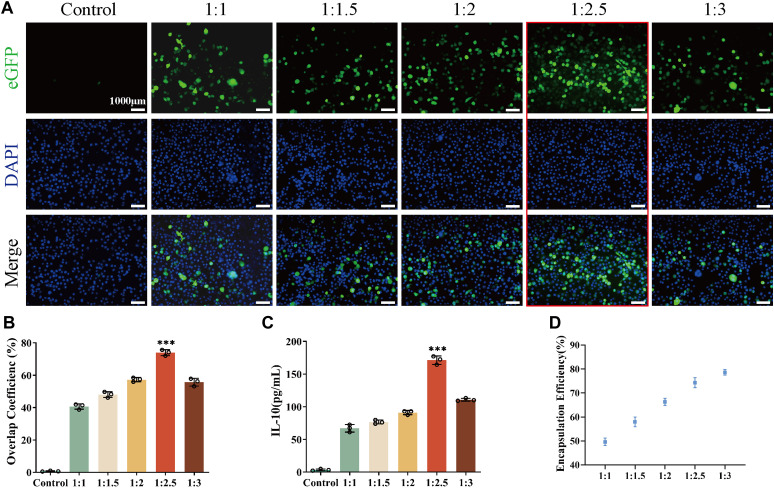
Transfection efficiency of pIL10-LNP under different ratios; **(A)** eGFP fluorescence expression 48 h after transfection; **(B)** Co-localization rate of eGFP fluorescence and nuclei 48 h after transfection; **(C)** Quantitative analysis of IL-10 secretion amount 48 h after transfection; **(D)** Encapsulation efficiency of pIL10-LNP under different ratios. *p < 0.05, **p < 0.01, ***p < 0.001 in one-way analysis of variance (ANOVA) and Tukey’s *post hoc* test.

It has been demonstrated that there is an optimal concentration ratio of liposome-carrying plasmid complexes during the cell transfection process (Marin et al., 2020; Suzuki et al., 2022). Therefore, to determine the ratio of pIL10 to LNPs for optimal transfection efficiency, five groups of pIL10-LNP were prepared (1:1, 1:1.5, 1:2, 1:2.5, and 1:3) and transfected into BMMs for 48 h. The colocalization rate of eGFP fluorescence and nuclei was observed (Figure 2A). The co-localization rate was highest at ratio 1:2.5 (Figure 2B). Whereas, the IL-10 secretion was highest at ratio 1:2.5 measured 48 h after transfection (Figure 2C), these were consistent with the results of fluorescence co-localization, suggesting that the optimal ratio of pIL10 and LNP was 1:2.5. The molecular weight of pDNA used in this study was 563 bp (Supplementary Table S2). The encapsulation efficiency of pIL10-LNP at different ratios was determined using ultrafiltration centrifuge tubes by the dialysis method (Figure 2D). The encapsulation efficiency increased with increasing pIL10 concentration. This may be because pIL10 has a slight negative charged and can be attracted to the positively charged LNPs by electrostatic adsorption. A higher encapsulation efficiency of more than 70% was found for pIL10-LNP at a ratio of 1:2.5. Combined with the fluorescence co-localization rate and the results of the IL-10 release test, pIL10-LNP at a ratio of 1:2.5 was selected for later tests.

**FIGURE 3:**
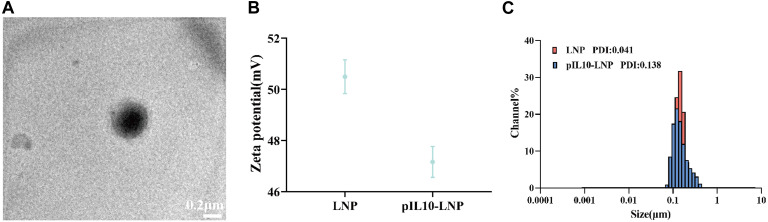
Characterization of pIL10-LNP. **(A)** TEM image of pIL10-LNP; **(B)** Zeta potential of unloaded LNP and pIL10-LNP; **(C)** Particle size and dispersity of unloaded LNP and pIL10-LNP.

As observed by transmission electron microscopy (TEM), pIL10-LNP was uniform in size, with regular spherical nanoparticles and a visible lipid bilayer structure (Figure 3A). The zeta potential (Figure 3B) and particle size distribution (Figure 3C) of pIL10-LNP showed that it had a slight decrease in the zeta potential compared to unloaded LNP. A possible reason for this is that the negatively charged pIL10 was electrostatically adsorbed onto the positively charged LNP. The mean particle sizes of unloaded LNP and pIL10-LNP were 131.1 nm and 136.3 nm, respectively, and the mean polymer dispersity indexes (PDI) were 0.041 and 0.138, respectively, suggesting that pIL10-LNP has a uniform dispersion and particle size.

### 3.2 Characterization of GEESs

**FIGURE 4:**
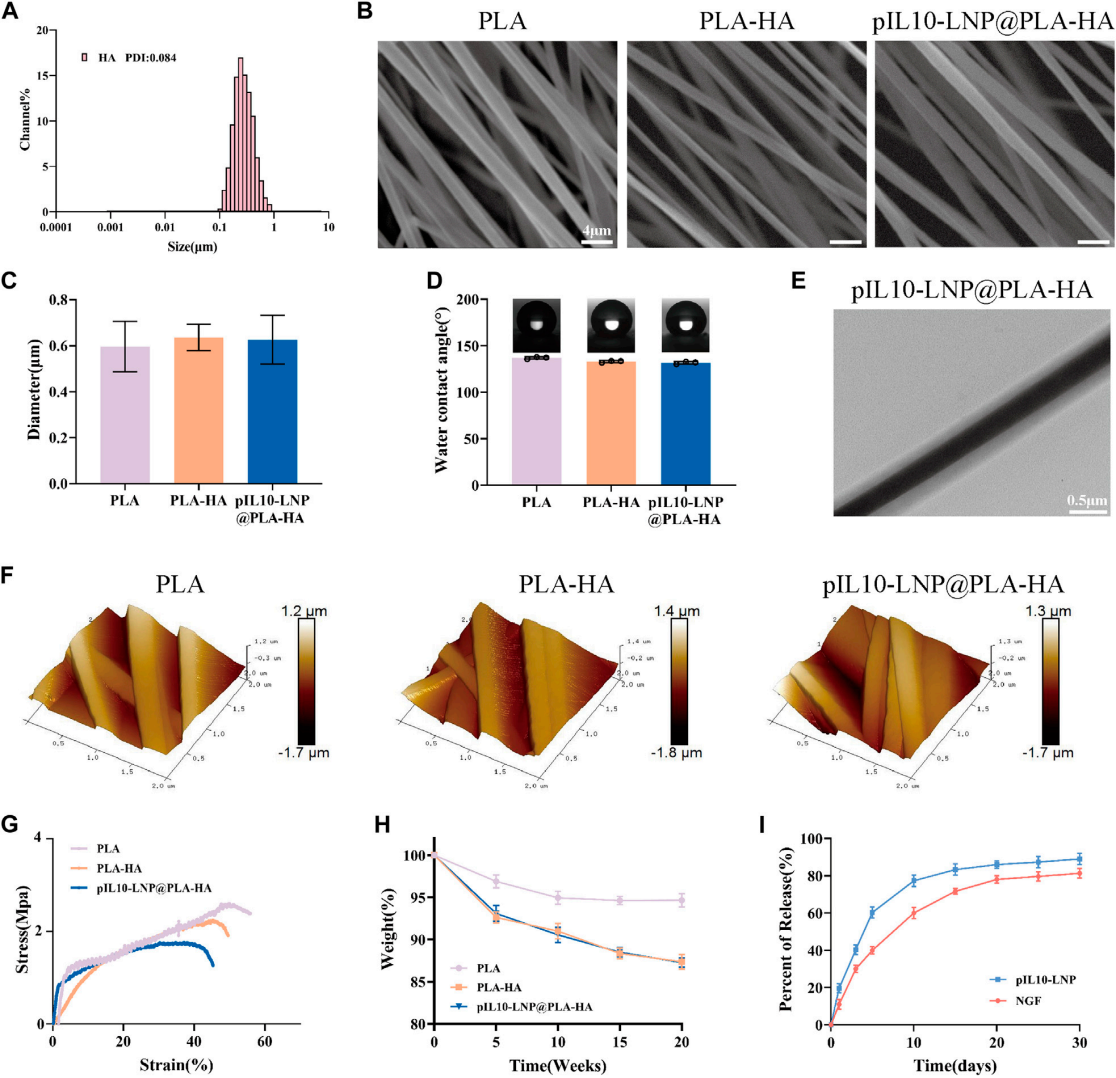
Characterization of genetically engineered electrospun scaffolds. **(A)** Assessment of particle size and PDI of HA particles loaded with β-NGF; **(B)** Morphology characterization of different electrospun scaffolds by SEM; **(C)** Particle size of fibers of electrospun scaffolds in each group; **(D)** WCA of different electrospun scaffolds; **(E)** Observation of the internal structure of scaffolds by TEM; **(F)** Ra characterization of different electrospun scaffolds; **(G)** Stress-strain curves of different scaffolds; **(H)** Degradation curves of different scaffolds; **(I)** pIL10-LNP and NGF release curves of pIL10-LNP@PLA-HA scaffolds.

Compared with traditional PLA electrospun fibers, microsol electrospun fibers have a unique internal core-shell structure. In this study, the “core” of the microsol electrospun scaffold consisted of HA; therefore, the NGF-loaded HA microsol particles in dichloromethane (DCM) were first detected to characterize their particle size and PDI using a dynamic light scattering particle size analyzer. As shown in Figure 4A, the mean particle size of the HA microsol particles was 264 nm, and their PDI was 0.084, suggesting that the HA microsol particles were uniformly and stably distributed. Because the oriented electrospun scaffold mimics the structure of the neural extracellular matrix, the oriented fiber scaffold can provide a good platform for the directional growth and connection of cells and promote the growth and extension of nerve cells, which is conducive to axonal growth and nerve tissue repair. Therefore, the surface morphology of the three groups of electrospun fiber scaffolds (PLA, PLA-HA, and pIL10-LNP@PLA-HA) was first observed using scanning electron microscopy (SEM). The results showed that the fiber scaffolds in each group were arranged in an oriented manner, had a smooth surface, and the direction of the fibers were highly consistent (Figure 4B). In addition, the fiber diameters in the three groups were detected using the ImageJ software and were 0.597 ± 0.11 μm, 0.637 ± 0.06 μm, and 0.627 ± 0.11 μm, respectively, and the difference was not statistically significant (*p* > 0.05) (Figure 4C). These results demonstrated that NGF and pIL10-LNP had no significant impact on the formation of fiber scaffolds, surface morphology, or fiber particle size.

The hydrophilic and hydrophobic properties of biological scaffolds determine whether cell adhesion is beneficial, thereby affecting cell proliferation, differentiation, tissue repair, and regeneration. Therefore, the water contact angle (WCA) of PLA, PLA-HA, and pIL10-LNP@PLA-HA groups were determined. As shown in Figure 4D, the WCA was (136.87 ± 1.14)°, (132.87 ± 1.10)°, and (131.63 ± 1.29)°, respectively, in the three groups. This is because, with the addition of HA, the scaffolds in the PLA-HA and pIL10-LNP@PLA-HA groups showed a slight increase in hydrophilicity compared to the PLA group, suggesting that the microsol electrospun scaffold possessed better cell adhesion.

The unique internal core-shell structure of microsol electrospun fibers is key to their ability to carry drugs and gene plasmids; therefore, the structure of the fibers in the three groups was detected and analyzed by TEM. The results showed that the core-shell structure of the scaffold in the pIL10-LNP@PLA-HA group showed a significant difference between lightness and darkness, and the outer PLA layer and the inner pIL10-LNP@HA structure could be clearly seen, but there were no visible nanoparticles under the microscope, probably because of the encapsulation of nanoparticles by HA (Figure 4E). Furthermore, the surface roughness (Ra) of the fibers in the three groups was observed using atomic force microscopy (AFM). As shown in Figure 4F, Ra was 272, 264, and 278 nm in the three groups, respectively, and there was no significant difference among the groups (*p* > 0.05), suggesting that the microsol electrospun filament was effective in encapsulating NGF and pIL10-LNP, and pIL10-LNP existed inside the core-shell structure.

The biological spine needs to bear the pressure and traction of surrounding tissues during daily activities owing to its special structure; therefore, the mechanical stress properties of the biological scaffold are highly important for stabilization in the injured area after implantation (Dos Santos Rodrigues et al., 2018). The mechanical properties of the fiber scaffolds in the three groups were first characterized by stress-strain tests. As shown in Figure 4G, the scaffolds in all three groups exhibited better tensile strength. However, the PLA-HA and pIL10-LNP@PLA-HA groups had slightly poorer mechanical properties than the PLA group, which might be attributed to the addition of HA. However, according to the results of a previous study (Xi et al., 2021), the mechanical properties of the scaffolds in the PLA-HA and pIL10-LNP@PLA-HA groups could still provide a good platform for cell and tissue growth. In addition, after providing support for tissue repair and regeneration, implanted biomaterials should degrade gradually to avoid hindering tissue regeneration; therefore, degradability is an important attribute of biomaterials. In the *in vitro* degradation study (Figure 4H), the weight of the scaffolds at each time point were measured. The degradation rates in the PLA-HA and pIL10-LNP@PLA-HA groups were higher than those in the PLA group. As shown in Figure 4I, the release rates of NGF and pIL10-LNP in the pIL10-LNP@PLA-HA group reached a high level on day 10 and then had a gradual release till day 30, with a total release rate above 80%. The scaffold in the pIL10-LNP@PLA-HA group released high levels of pIL10-LNP approximately 1 week into the acute phase of SCI to regulate the acute inflammatory response and immune response for a long duration. Nerve growth factor also had a stable release for a long period that benefited post-SCI nerve repair. Combined with the detection results of the scaffold degradation rate, it can be confirmed that the GEESs prepared satisfy the slow-release and degradable properties of tissue-engineered scaffolds.

### 3.3 Biocompatibility of GEESs

To assess the biocompatibility of the GEES, cell survival and proliferation on the scaffolds were first assessed *in vitro* using live-dead cell staining and the CCK-8 assay, respectively. Live/dead cell staining and semi-quantitative fluorescence analysis revealed no statistically significant differences among the groups (Supplementary Figures S1A, B) (*p* > 0.05), demonstrating that the layer-by-layer assembly of NGF-loaded HA hydrogels and pIL10-LNP did not alter the biocompatibility of the PLA electrospun filaments. Consistent results were obtained using the CCK-8 assay (Supplementary Figure S1C), but there was no statistically significant difference among the groups (*p* > 0.05), suggesting that the GEESs possessed good biocompatibility.

The fiber scaffolds in each group were then implanted subcutaneously into rats to further assess the biocompatibility of the materials *in vivo*. Hematoxylin-eosin (H&E) staining was performed 2 weeks after implantation (Supplementary Figure S2A). The inflammatory response was milder in the pIL10-LNP@PLA-HA group than in the other groups. The areas of the inflammatory zones were quantitatively analyzed using ImageJ software. The results showed that the number of inflammatory cells per unit area in pIL10-LNP@PLA-HA group was approximately 1,464.4 ± 146.5 mm^2^, which was smaller than that of the other groups (*p* < 0.05) (Supplementary Figure S2B). The results confirmed that the GEESs prepared had better biocompatibility and low immunogenicity and thus could be used for further tests.

### 3.4 Cell adhesion on GEESs

The strength of cell adhesion to a scaffold is critical for subsequent cell proliferation, differentiation, and tissue repair. Therefore, integrin β1, a transmembrane receptor protein involved in mediating signaling between the extracellular matrix and the cytoskeleton, was used to assess the cell adhesion status on the scaffolds in each group (Ahadian et al., 2018). The results showed that the morphology of the NSCs on the scaffolds was influenced by the structure of the oriented electrospun membrane, which exhibited a consistent orientation (Supplementary Figure S3A). According to the semi-quantitative fluorescence analysis (Supplementary Figure S3B), the green fluorescence intensity of integrin β1 had no statistically significant difference among the groups (*p* > 0.05), proving that the scaffolds in each group can provide a good platform for cell adhesion. In addition, consistent results were observed using SEM; the cells in each group had a consistent orientation with the electrospun membrane, the pseudopods were fully extended, and the cells were in a good state of extension (Supplementary Figure S3C).

### 3.5 GEESs promoted macrophage polarization

To assess the ability of genetically engineered electrospun scaffolds to modulate macrophage immune function, we co-cultured the scaffolds with BMMs for 3 and 5 days and assessed the expression of inflammatory genes in BMMs using qRT-PCR. The results (Supplementary Figures S4A, B) demonstrated a gradual decrease in the expression level of the pro-inflammatory gene TNF-α over time in the pIL10-LNP@PLA-HA group, accompanied by a concomitant increase in the expression level of the anti-inflammatory gene IL-10, which was significantly different compared to other groups (*p* < 0.05). Furthermore, we quantified the concentrations of IL-10 and TNF-α factors in the co-cultured supernatant. Consistently, the results (Supplementary Figures S4C, D) mirrored the trends observed in qRT-PCR, indicating that pIL10-LNP@PLA-HA significantly enhanced IL-10 secretion and suppressed TNF-α secretion in BMMs. These findings underscore the role of pIL10-LNP@PLA-HA in modulating the anti-inflammatory function of BMMs. By promoting the expression of the anti-inflammatory IL-10 gene through transfection, it facilitates the gradual release of IL-10 factor while progressively inhibiting the expression of inflammatory genes and cytokine release.

**FIGURE 5:**
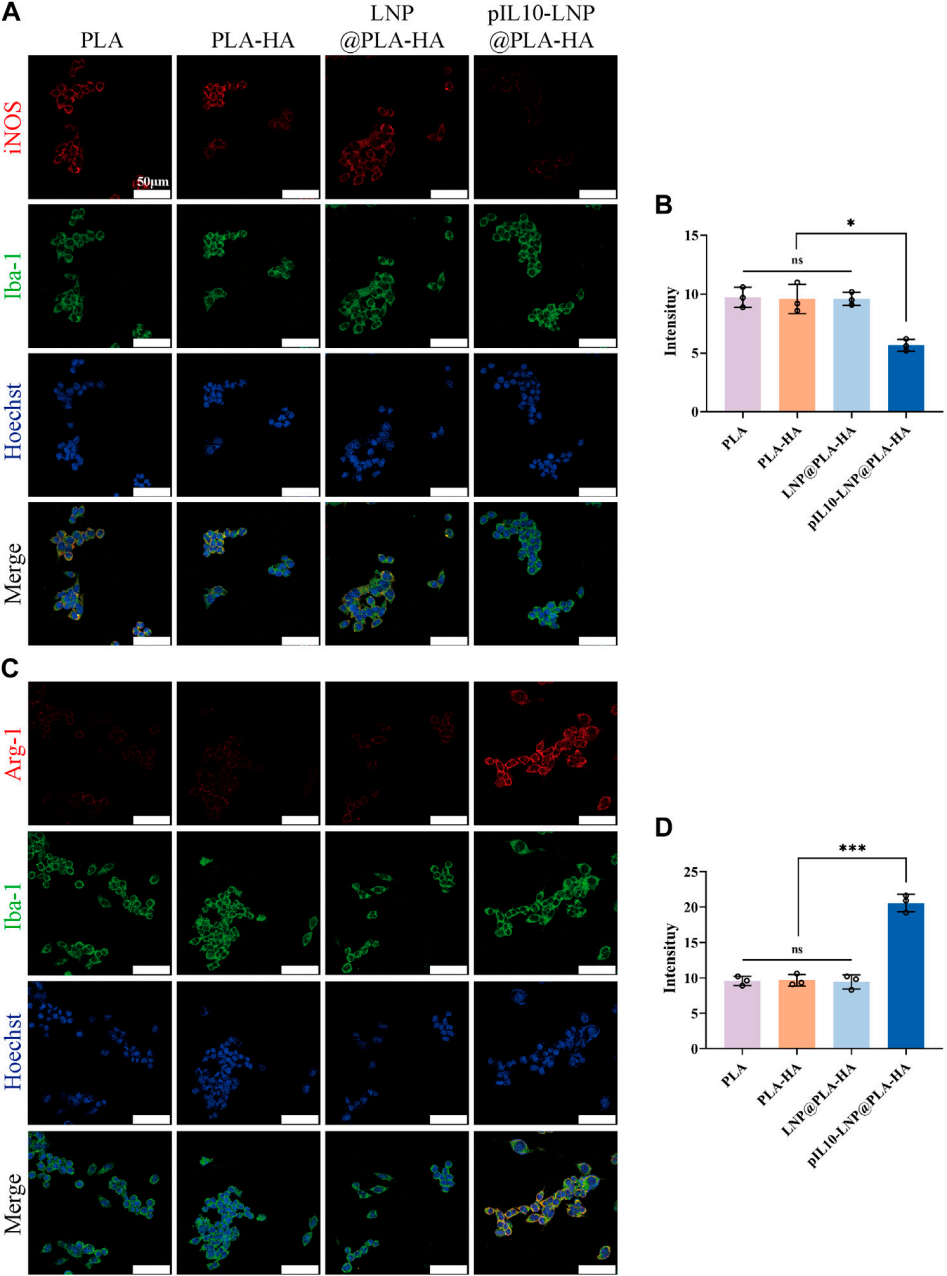
IF staining assessment of electrospun scaffolds regulating the subtypes of BMMs in each group. **(A)** IF labeling by Iba-1 (a macrophage marker, green fluorescence) and iNOS (a M1 macrophage marker, red fluorescence); **(B)** Fluorescence semi-quantitative analysis of M1 macrophage marker iNOS; **(C)** IF labeling by Iba-1 (a macrophage marker, green fluorescence) and Arg-1 (a M2 macrophage marker, red fluorescence); **(D)** Fluorescence semi-quantitative analysis of M2 macrophage marker Arg-1. *p < 0.05, **p < 0.01, ***p < 0.001 in one-way ANOVA and Tukey’s *post hoc* test.

The regulatory effect of the electrospun scaffolds on macrophage polarization was assessed *in vitro*. Rat BMMs are classified into the pro-inflammatory M1 and anti-inflammatory M2 types. Macrophages were first labeled with Iba-1, whereas M1 and M2 macrophages were specifically labeled with inducible nitric oxide synthase (iNOS) and arginase-1 (Arg-1), respectively. Immunofluorescence (IF) microscopy images and semi-quantitative fluorescence analysis showed that the fluorescence intensity of iNOS in the pIL10-LNP@PLA-HA group was significantly lower than that in the other groups (*p* < 0.05) (Figures 5A, B), while the fluorescence intensity of Arg-1 was significantly higher than that of the other groups (*p* < 0.05) (Figures 5C, D). These findings demonstrated that GEESs can effectively induce M2 polarization of BMMs and inhibit M1 polarization in *in vitro* cell cultures, with better immunoregulatory properties (Chung and Son, 2014).

### 3.6 GEESs enhanced nerve regeneration *in vitro*

**FIGURE 6:**
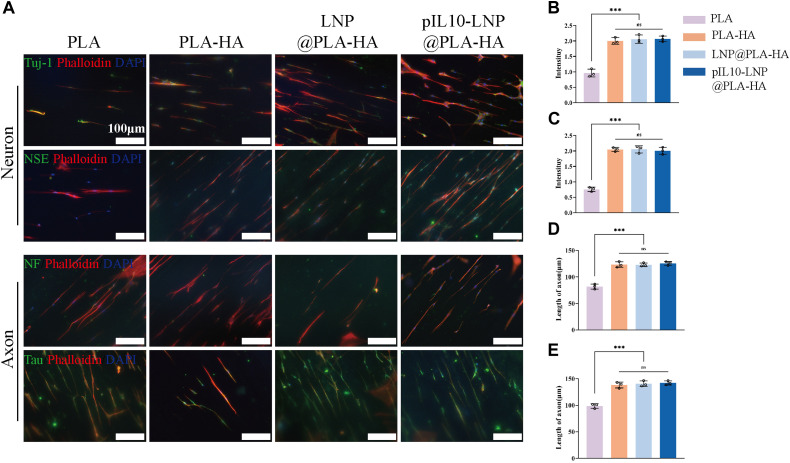
IF staining by neuron-specific markers of NSCs on different electrospun scaffolds. **(A)** IF staining (green fluorescence) of Tuj-1, NSE, NF, and Tau proteins after differentiation of NSCs into neurons on different electrospun scaffolds; **(B–E)** Fluorescence semi-quantitative analysis of Tuj-1, NSE and quantification of axon length (NF, Tau). *p < 0.05, **p < 0.01, ***p < 0.001 in one-way ANOVA and Tukey’s *post hoc* test.

Many NSCs in the CNS that differentiate into neuronal cells are important for nerve tissue repair. The fiber scaffolds were co-cultured with NSCs, and their potential to induce NSCs to differentiate into neurons was assessed using four indicators: neurofilament proteins (NF), neuron-specific enolase (NSE), neuronal microtubule-associated protein (Tau protein), and microtubule-associated protein for neuron-specific differentiation (Tuj-1). As shown in the IF microscopic images, the cell morphology in each group was oriented along the fiber direction. When NGF was loaded into the PLA-HA, LNP@PLA-HA, and pIL10-LNP@PLA-HA groups, NSCs displayed a trend of differentiation toward neuronal cells in each group (Figure 6A). Moreover, the results of fluorescence semi-quantitative analysis of the above Tuj-1 and NSE showed that the fluorescence intensity in PLA-HA, LNP@PLA-HA, and pIL10-LNP@PLA-HA groups was significantly higher than those in the PLA group, and the differences were statistically significant. Moreover, our findings indicate that the incorporation of NGF in the PLA-HA, LNP@PLA-HA, and pIL10-LNP@PLA-HA groups markedly enhanced the elongation of axonal fibers in nerve cells, thereby facilitating the establishment of neural networks (*p* < 0.05) (Figures 6B–E). In summary, NGF loaded into scaffolds can be slowly released to continuously promote the differentiation of NSCs into neuronal cells, which benefits SCI repair.

### 3.7 GEESs improved post-SCI motor function and pathology in rats

Rat T9 hemi-transverse SCI models were established, and samples were taken at 1, 4, and 8 weeks postoperatively (Supplementary Figure S5A). The motor function recovery of the rats in each group was assessed using the Basso-Beattie-Bresnahan (BBB) (Supplementary Figure S5B) and inclined plane test (IPT) (Supplementary Figure S5C) scores. The results showed that the BBB scores of both the SCI and PLA groups were <5 points at 8 weeks postoperatively, indicating that the rats had limited nerve self-healing ability after SCI. The pIL10-LNP@PLA-HA group had a postoperative BBB score of 5.5 ± 0.84 and an IPT score of 46.67 ± 6.06 at 3 and 4 weeks, respectively, which had statistically significant differences from the other groups. At 8 weeks, the BBB score and IPT score of pIL10-LNP@PLA-HA group were 13 ± 1.26 and 58.33 ± 4.08 points, respectively, suggesting weight-bearing mobility and coordinated fore- and hind-limb movements. It can be concluded that the GEESs can contribute to the early motor functional recovery of rats after SCI.

Furthermore, the post-SCI pathology of the rats in each group was assessed using H&E staining. As shown in Supplementary Figure S6A, a right hemi-transverse spinal cord defect was observed in all groups and spinal cord cavities appeared at the injury site, suggesting successful modeling. No significant change was observed in the cavity area in the SCI group at 4 and 8 weeks postoperatively, and the difference was not statistically significant (*p* > 0.05), further illustrating the limited self-healing ability of rats after SCI. The NGF added to the PLA-HA and LNP@PLA-HA groups promoted nerve repair and reduced the cavity area after SCI. However, the cavity area was significantly smaller in the pIL10-LNP@PLA-HA group than in the other groups at four and 8 weeks postoperatively (*p* < 0.05) (Supplementary Figures S6B, C) and the lesion area was the smallest. This may be that pIL10-LNP was sustainably released by electrospun filaments, which play an immunoregulatory role, creating a favorable microenvironment for nerve repair.

### 3.8 GEESs modulated post-SCI inflammatory response in rats

**FIGURE 7:**
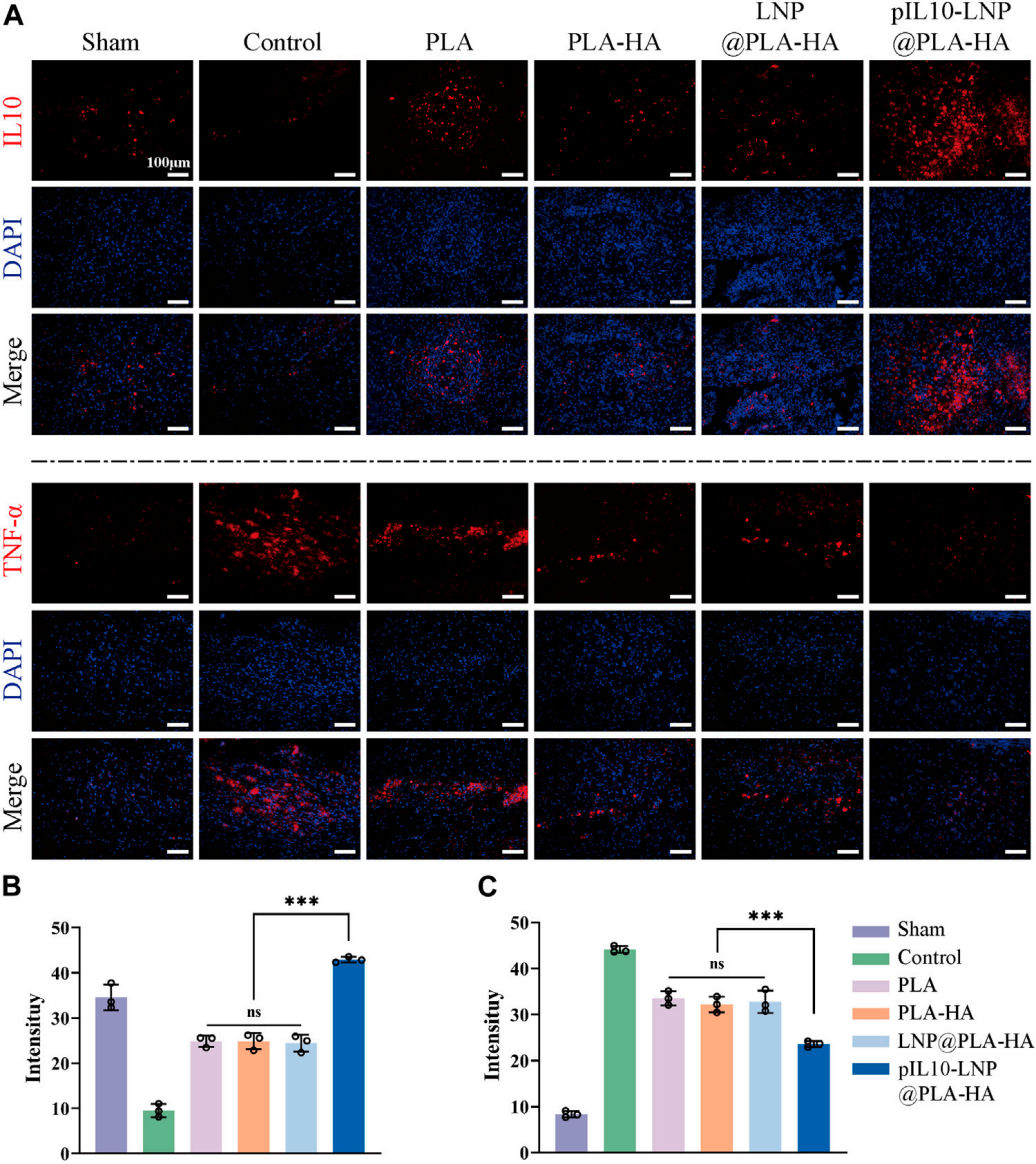
Assessment of immunoregulatory function of electrospun scaffolds in each group at 7 days postoperatively. **(A)** IF labeling of IL-10 and TNF-α; **(B–C)** Fluorescence semi-quantitative analysis of IL-10 and TNF-α. *p < 0.05, **p < 0.01, ***p < 0.001 in one-way ANOVA and Tukey’s *post hoc* test.

The inflammatory cells activated after SCI releases pro-inflammatory cytokines for secondary injury, which can lead to a series of cascade reactions, thereby worsening the injury, and affecting nerve tissue repair. Therefore, it is crucial to assess the early anti-inflammatory effect of composite fiber scaffolds *in vivo*. Since GEESs can transfect local tissues with pIL10 to promote the secretion of IL-10 at the injury site and the M1 macrophage marker TNF-α were labeled by IF to reflect the immunoregulation status at the injury site (Figure 7A). The results revealed that the fluorescence intensity of IL-10 in pIL10-LNP@PLA-HA group was significantly higher than that in other groups, while the fluorescence intensity of TNF-α in pIL10-LNP@PLA-HA group was significantly lower than that of the other groups, with statistically significant differences (*p* < 0.05) (Figures 7B, C).

**FIGURE 8:**
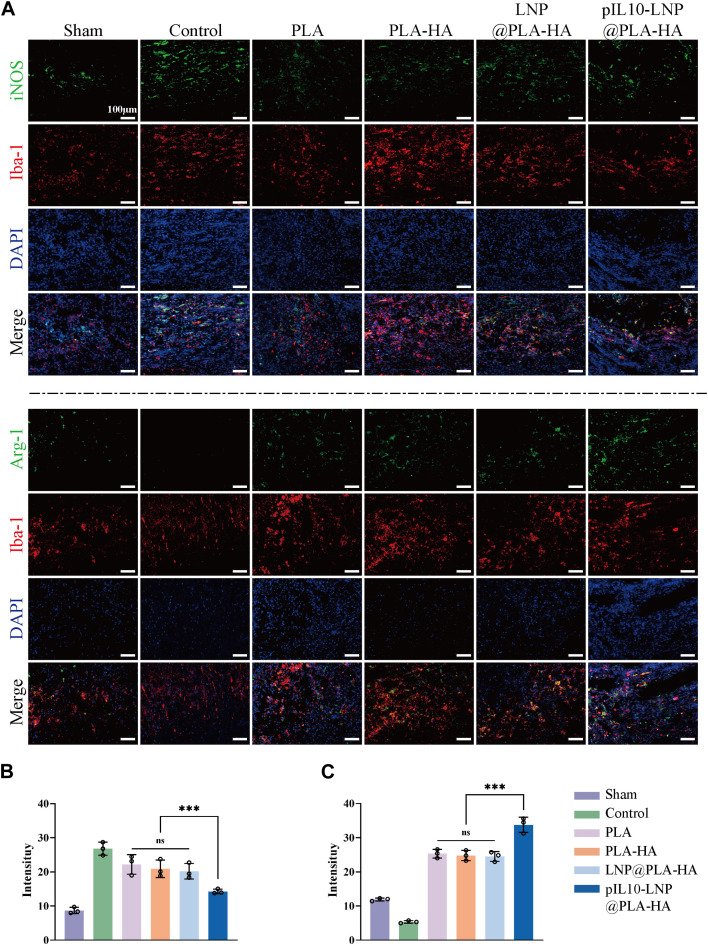
Assessment of immunoregulatory function of electrospun scaffolds in each group at 7 days postoperatively. **(A)** Macrophage marker Iba-1 (red fluorescence), and M1 and M2 macrophage markers iNOS and Arg-1 (green fluorescence); **(B–C)** Fluorescence semi-quantitative analysis of iNOS and Arg-1. *p < 0.05, **p < 0.01, ***p < 0.001 in one-way ANOVA and Tukey’s *post hoc* test.

To further clarify macrophage polarization at the injury site, macrophages at the injury site were labeled with Iba-1, whereas M1 and M2 macrophages were specifically labeled with iNOS and Arg-1, respectively (Figure 8A). The results showed aggregation of more macrophages/microglia at the injury site in the SCI and the other control groups than in the sham group. The IF imaging and semi-quantitative analysis revealed that the fluorescence intensity of iNOS in the pIL10-LNP@PLA-HA group was significantly lower than that of the other groups (*p* < 0.05), while the fluorescence intensity of Arg-1 was significantly higher than that of the other groups (*p* < 0.05) (Figures 8B, C).

**FIGURE 9:**
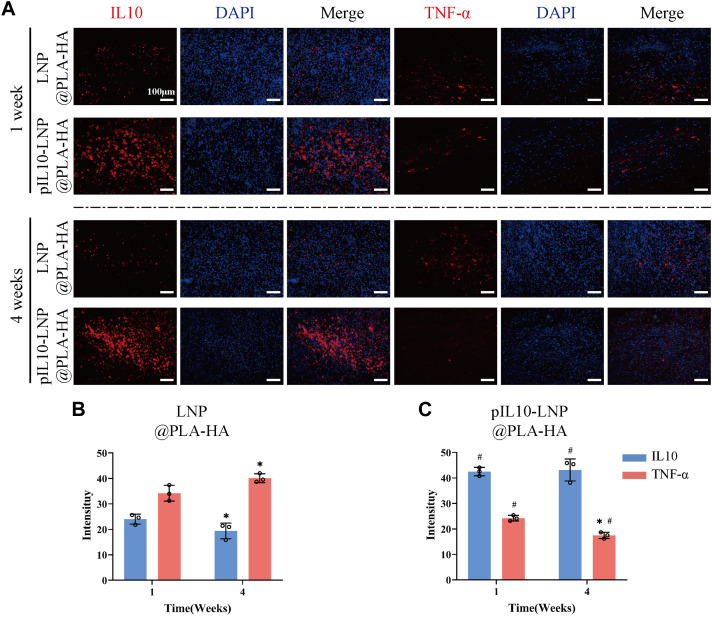
Assessment of immunoregulatory function of LNP@PLA-HA and pIL10-LNP@PLA-HA electrospun scaffolds at 4 weeks postoperatively. **(A)** IL-10 and TNF-α IF labeling assays at one and 4 weeks postoperatively; **(B–C)** Comparison of fluorescence semi-quantitative analysis results of IL-10 and TNF-α on LNP@PLA-HA and pIL10-LNP@PLA-HA scaffolds at one and 4 weeks postoperatively. *p < 0.05, **p < 0.01, ***p < 0.001 by one-way ANOVA and Tukey’s *post hoc* test, #*p* indicates a significant difference between LNP@PLA-HA and pIL10-LNP@PLA-HA.

pIL10-LNP@PLA-HA stably released pIL10-LNP for 30 d, suggesting that it may have a regulatory effect on chronic post-SCI inflammation. Therefore, IL-10 and TNF-α IF labeling was performed on samples from the LNP@PLA-HA and pIL10-LNP@PLA-HA group at 4 weeks after SCI (Figure 9A). The results showed a decrease in fluorescence intensity of IL-10 and an increase of TNF-α in LNP@PLA-HA group at 4 weeks after SCI compared with those at 7 days after SCI (Figure 9B). In pIL10-LNP@PLA-HA group, the fluorescence intensity of IL-10 had no significant changes, while the fluorescence intensity of TNF-α significantly declined at 4 weeks after SCI compared with those at 7 days (Figure 9C). It can be inferred that the pIL10-LNP@PLA-HA scaffold could release pIL10-LNP for a long time and induce higher levels of IL-10 at the injury site, thereby attenuating the inflammatory response.

**FIGURE 10:**
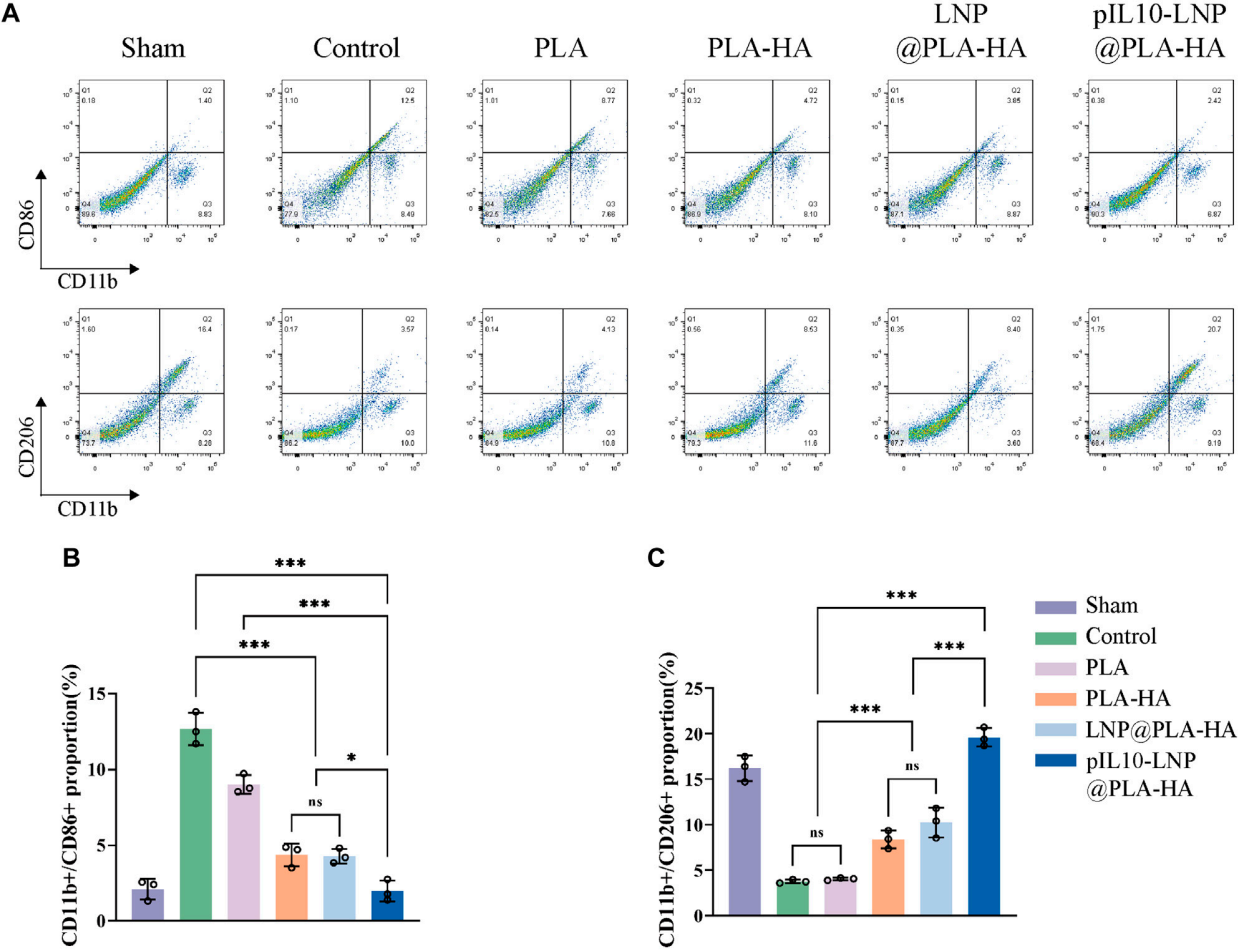
Flow cytometry of spinal cord samples in each group at 7 days postoperatively. **(A)** Flow cytometry of proportion of CD11b/CD86-positive cells, and CD11b/CD206-positive cells; **(B)** Statistical analysis of CD11b/CD86 positive rates; **(C)** Statistical analysis of CD11b/CD206 positive rates. *p < 0.05, **p < 0.01, ***p < 0.001 in one-way ANOVA and Tukey’s *post hoc* test.

Furthermore, the role of the composite fiber scaffolds in regulating macrophage subtypes 7 days after SCI was assessed *in vivo* using flow cytometry. CD206 was used to label macrophages, and CD86 and CD206 were used to label M1 and M2 macrophages, respectively (Figure 10A). The results showed that the proportions of CD11b/CD86 double-positive macrophages was lower in the PLA group than in the control group (*p* < 0.05); however, the changes in the proportion of CD11b/CD206 double-positive macrophages were not significant. Microsol electrospun scaffolds without loaded target genes (PLA-HA and LNP@PLA-HA groups) decreased and increased the proportion of M1 and M2, respectively, owing to the inhibition of migration, chemotaxis, and proliferation of lymphocytes with the introduction of HA, which maintained the homeostasis of tissues at the injury site (Moshayedi and Carmichel, 2013; Frischknecht et al., 2014; Gupta et al., 2019). The GEESs slowly released pIL10-LNP to significantly induce M2 polarization of macrophages at the injury site, thus significantly reducing the proportion of M1 macrophages, with a significant difference compared to other groups (*p* < 0.05) (Figures 10B, C). In summary, GEESs can effectively modulate the immune cell subtypes during the acute phase of SCI.

### 3.9 GEESs modulated post-SCI inflammatory response in rats

**FIGURE 11:**
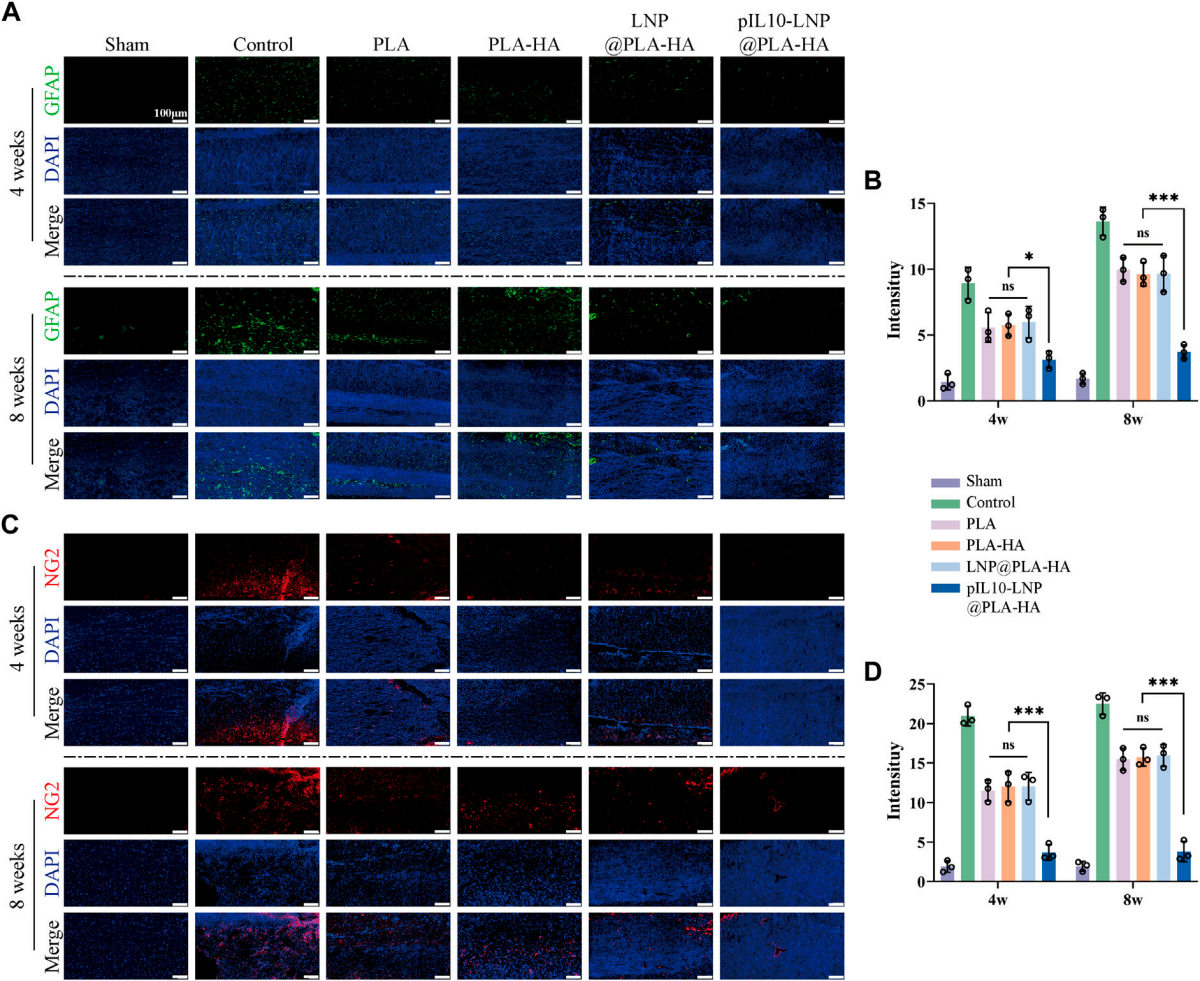
Assessment of astrocyte activation and glial scar formation regulated by electrospun scaffolds. **(A)** GFAP fluorescence staining (green fluorescence) of spinal cord samples in each group at four and 8 weeks postoperatively; **(B)** GFAP fluorescence semi-quantitative analysis; **(C)** NG2 fluorescence staining (red fluorescence) of spinal cord samples in each group at 4 and 8 weeks postoperatively; **(D)** NG2 fluorescence semi-quantitative analysis. *p < 0.05, **p < 0.01, ***p < 0.001 in one-way ANOVA and Tukey’s *post hoc* test.

The role of the composite fiber scaffolds in post-SCI nerve tissue repair and regeneration *in vivo* was assessed based on neuronal regeneration, axonal sprouting, astrocyte activation, and glial scar regeneration. The unique aggregation of astrocytes in the central nervous system is activated and promoted after SCI, and dense glial scars are formed in an inflammatory environment to fill the post-SCI cavity, which becomes an obstacle to nerve and axon regeneration (Tohda and Kuboyama, 2011; Ziegler et al., 2018; Masterman and Ahmed, 2021). Therefore, GFAP and NG2 were first used to label the activated astrocytes and glial scar tissues, respectively, at the site of injury. As shown in Figures 11A, C, no significant differences were observed among the PLA, PLA-HA, and LNP@PLA-HA groups at 4 and 8 weeks (*p* > 0.05); however, the number of activated astrocytes and glial scar tissues was significantly lower in the pIL10-LNP@PLA-HA group than in the other groups at 4 and 8 weeks. Moreover, semi-quantitative fluorescence analysis showed that the pIL10-LNP@PLA-HA group had a lower fluorescence intensity of activated astrocytes and glial scar tissues than the other groups (*p* < 0.05) (Figures 11B, D), suggesting that GEESs may reduce local scar tissue formation by attenuating the post-SCI inflammatory response.

**FIGURE 12:**
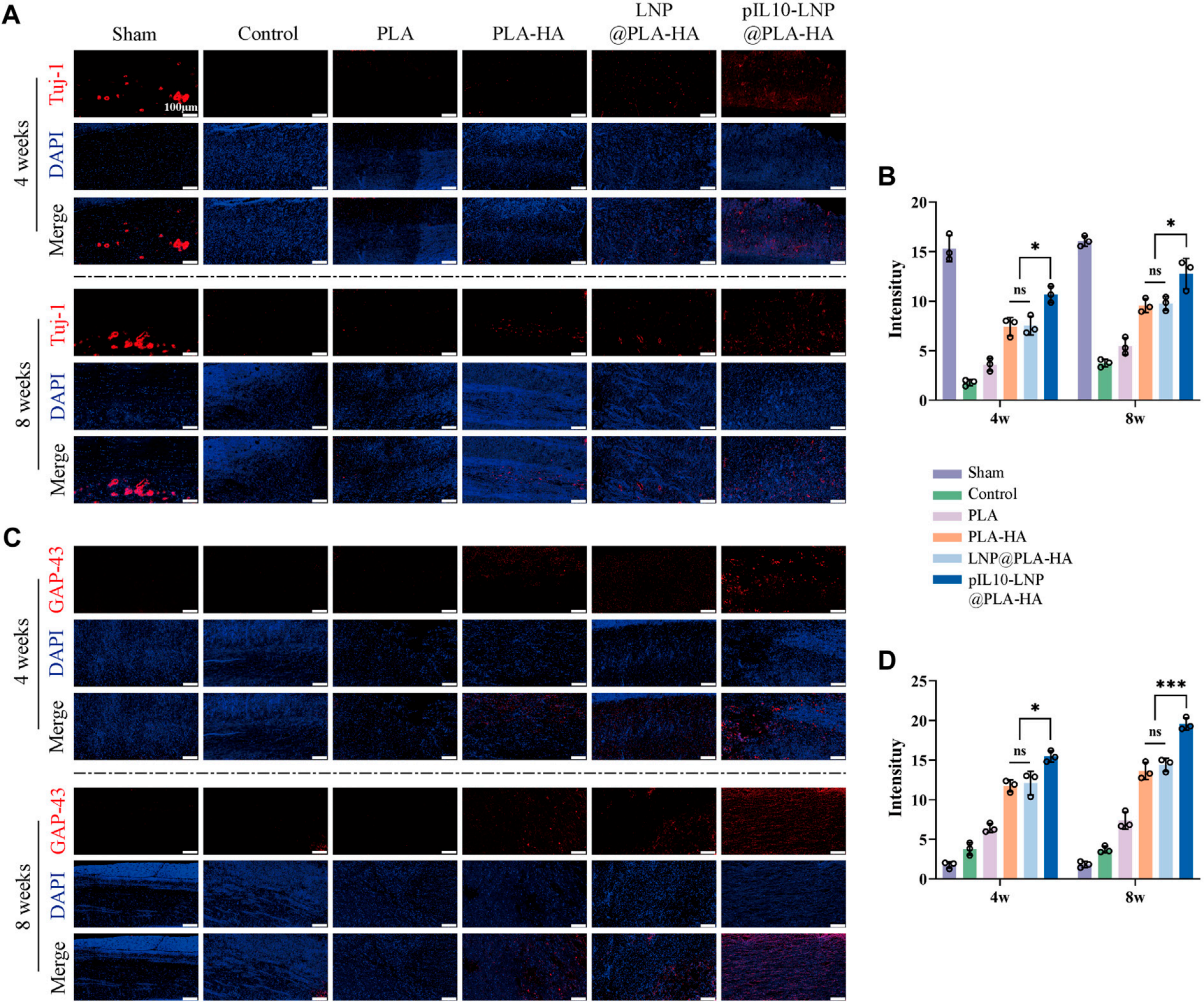
Assessment of neuronal growth and axonal sprouting regulated by electrospun scaffolds. **(A)** Tuj-1 fluorescence staining (red fluorescence) of spinal cord samples in each group at 4 and 8 weeks postoperatively; **(B)** Tuj-1 fluorescence semi-quantitative analysis; **(C)** GAP-43 fluorescence staining (red fluorescence) of spinal cord samples in each group at four and 8 weeks postoperatively; **(D)** GAP-43 fluorescence semi-quantitative analysis. *p < 0.05, **p < 0.01, ***p < 0.001 in one-way ANOVA and Tukey’s *post hoc* test.

Furthermore, newly differentiated neurons and axonal sprouts were labeled with Tuj-1 and GAP-43 (one of the indicators for nerve growth and coordinated axon extension, respectively) to assess the ability of the composite fiber scaffolds to enhance nerve regeneration. As shown in Figure 12A, 4 and 8 weeks after SCI, more Tuj-1-positive neuronal cells were observed at the injury site in the PLA-HA, LNP@PLA-HA, and pIL10-LNP@PLA-HA groups than in the SCI and PLA groups. Semi-quantitative fluorescence analysis revealed that the intensity of Tuj-1 was the highest in the pIL10-LNP@PLA-HA group, and the difference was statistically significant (*p* < 0.01) (Figure 12B). Consistent results were obtained with GAP-43 labeling (Figure 12C); that is, a large number of axonal sprouts could be seen at the injury site in the pIL10-LNP@PLA-HA group at 4 and 8 weeks after SCI, and fluorescence semi-quantitative analysis showed a higher fluorescence intensity of GAP-43 than other groups (*p* < 0.05) (Figure 12D). It can be concluded that the GEESs provide a good platform for nerve tissue repair and enhance nerve repair and regeneration by inhibiting acute inflammatory responses and attenuating glial scar formation.

## 4 Discussion

The inflammatory response, glial scar regeneration, and difficulties in nerve and axon regeneration are the three most difficult problems in SCI treatment (Assinck et al., 2017; Freund et al., 2019). In SCI, an intense local inflammatory response exacerbates the aggregation of reactive astrocytes and dense glial scars are formed to fill the post-SCI cavity, thus hindering neuronal regeneration and axonal sprouting. Therefore, it is necessary to manage both immunoregulation and nerve repair during SCI treatment. In this study, an electrospinning system was developed for efficient, stable, and long-lasting delivery of genetic materials and drugs. In this system, pIL10 was used as the genetic information material to promote the release of an anti-inflammatory factor (IL-10) in local tissues after SCI, thus alleviating the inflammatory response during the acute phase of SCI and regulating long-term chronic inflammation after SCI, which aimed to provide a favorable microenvironment for nerve tissue repair and regeneration. Meanwhile, the slow release of NGF facilitated the differentiation of NSCs into neuronal cells and axonal growth and promoted nerve tissue repair. The results showed that the prepared microsol electrospun scaffolds could release pIL10-LNP stably for a long time. The *in vitro* and *in vivo* experiments indicated that the cells and tissues could be transfected with pIL10-LNP released from the composite fiber scaffolds, which successfully released IL-10. In addition, *in vitro* experiments showed that the expression of iNOS in the pIL10-LNP@PLA-HA group was substantially lower, whereas Arg-1 was higher, than those in the other groups. The expression of the neuronal and axonal marker proteins Tuj-1, NSE, NF, and Tau was considerably increased in the pIL10-LNP@PLA-HA group compared to that in the other groups. Moreover, the release of IL-10 at the injury site was induced in a long-term and stable manner in the pIL10-LNP@PLA-HA group to regulate the anti-inflammatory/pro-inflammatory imbalance in the acute phase of SCI, creating a favorable environment for the slow-release of NGF, thereby promoting nerve repair. Electrospun scaffolds contribute to functional motor recovery after SCI via immunoregulation and nerve repair.

IL-10, a pleiotropic cytokine with potent anti-inflammatory properties, is produced by almost all leukocyte subpopulations, with macrophages being the main source. IL-10 can inhibit the expression of inflammatory factors, such as TNF-α, IL-6, and IL-1, via activated M2 macrophages, attenuating the inflammatory response (Hedrich and Bream, 2010; Saraiva and OGarra, 2010; Glocker et al., 2011; Devkota et al., 2012; Mannino et al., 2015). It has been demonstrated that IL-10-knockout mice have significant inflammatory cell infiltration, elevated expressions of related inflammatory factors, and enhanced immune responses (Kühn et al., 1993; Begue et al., 2011; Shouval et al., 2014; Zigmond et al., 2014), suggesting that IL-10 plays a pivotal role in immunoregulation. In a previous study, we found that the expression level of IL-10 in local tissues after SCI was substantially lower than in healthy controls (Xi et al., 2021). In addition, it has been shown that IL-10 is involved in the metabolic remodeling of macrophages, i.e., it inhibits the expression of mTORC1 through the IL-10/STAT3/DDIT4 axis, thus restraining the mTOR signaling pathway, suppressing the lipopolysaccharide-activated macrophage glycolysis, and enhancing the oxidative phosphorylation of macrophages. In the absence of IL-10, NLRP3 in damaged mitochondria accumulated in macrophages will be abnormally activated, and the resulting IL-1β will amplify the immuno-inflammatory response (Ip et al., 2017). Therefore, IL-10 gene therapy for immunoregulation is a promising therapeutic approach for SCI.

Maintaining the activity of the target gene *in vivo* is critical for the success of gene therapy. Successfully transfected cells can continue to secrete the target protein for immunoregulation (Raftery et al., 2016; Zha et al., 2020). However, the commonly used delivery methods for gene vectors are characterized by the rapid loss of vectors, easy degradation, and poor transfection efficiency (Shafiee and Atala, 2017). Our research group has previously successfully grafted cationic liposomes onto microsol electrospun filaments, which can release liposomes at an early stage of injury in response to the local acidic microenvironment after SCI (Xi et al., 2021). Although a severe inflammatory response usually occurs in the early stages of SCI, causing a series of secondary injuries, the post-SCI inflammatory response is a continuous process and long-term chronic inflammation may cause persistent tissue damage and nerve non-healing. To solve this problem, pIL10 was encapsulated by LNP using the microfluidic technique, whose particle size was smaller than that of the electrospun fibers, and pIL10-LNP were further encapsulated by HA. Owing to the different electron permeabilities of PLA and HA, the sol particles were stretched under the action of an electric field, and the solvent evaporation rates of the two were different; thus, PLA formed a core-shell structure encapsulating HA in the outer layer more quickly than HA, based on which the pIL10-LNP-loaded microsol electrospun fiber scaffolds were prepared in this study. Compared with the previous system, HA maintained DNA bioactivity without denaturation during the encapsulation of pIL10-LNP into electrospun PLA filaments. The release rate of pIL10-LNP on day 1 (20%) was lower than that of the previous system (80%), but it reached approximately 70% during the acute phase of SCI (7 days) and lasted for up to 30 days, with a total release rate of up to 90%. *In vivo* experiments demonstrated that the expression of TNF-α at 4 weeks after SCI in pIL10-LNP@PLA-HA group significantly declined compared with that at 7 days after SCI, while there was no significant difference in the expression of IL-10 at the two time points, suggesting that the pIL10-LNP@PLA-HA scaffolds can transfect tissues at the injury site stably for a long time to secrete IL-10 for immunoregulation, effectively suppressing the inflammatory response in both acute and chronic phases of SCI. In addition, slow-release NGF facilitates the differentiation of NSCs into neuronal cells and axonal growth *in vivo* and *in vitro*, reduces glial scar formation at the injury site, and promotes nerve repair and regeneration, thereby ameliorating motor function in rats.

In conclusion, GEESs were prepared using microfluidic and microsol electrospinning techniques. It was found that HA could effectively encapsulate and protect pIL10-LNP into PLA electrospun filaments, and the core-shell structure formed could realize the long-term slow release of pIL10-LNP with good bioactivity, displaying good biocompatibility. The pIL10-LNP@PLA-HA scaffolds not only relieved the severe inflammatory response during the acute phase of SCI, but also regulated post-SCI chronic inflammation for a long time, creating a long-term stable and good microenvironment for neuron and axon regeneration. With the addition of NGF, the differentiation of NSCs into neuronal cells and axonal sprouting were both further facilitated, the activation and aggregation of astrocytes were inhibited, and glial scar formation was reduced, thereby improving motor functional recovery after SCI. This study provides a safe and potentially new strategy for gene + drug treatment of SCI.

## 5 Conclusion

In conclusion, this study, a novel composite drug delivery system, pIL10-LNP@PLA-HA, was successfully constructed that could stably release pIL10-LNP and NGF for a long time to ameliorate the post-SCI inflammatory response and enhance nerve repair. This system has good biocompatibility, promotes cell adhesion and proliferation, and effectively induces the M2 polarization of macrophages and secretion of anti-inflammatory factors for a long time, thereby improving the post-SCI inflammatory response. In addition, the slow release of NGF facilitates the differentiation of NSCs into neuronal cells and promotes nerve repair. In summary, pIL10-LNP@PLA-HA effectively handled two major issues (immunoregulation and nerve repair) in SCI treatment, providing a new strategy for gene + drug treatment of SCI.

## Data Availability

The original contributions presented in the study are included in the article/Supplementary Material, further inquiries can be directed to the corresponding authors.
