# Supplementary material for: Genetically engineered electrospinning contributes to spinal cord injury repair by regulating the immune microenvironment
Source: Front Bioeng Biotechnol. 2024 Jun 12;12:1415527. doi: 10.3389/fbioe.2024.1415527 (PMC11199540; doi:10.3389/fbioe.2024.1415527)
Supplement: Supplementary file 1 [file Presentation1.zip › Supplementary_Material/Supplementary_Material.docx]

Supplementary Material

# Supplementary Tables

**Supplementary Table S1.** Primer sequences of each gene

| Target | Forward | Reverse |
| --- | --- | --- |
| IL-10 | GCTCAGCACTGCTATGTTGC | TTGTCACCCCGGATGGAATG |
| TNF-α | ACTGAACTTCGGGGTGATCG | GCTTGGTGGTTTGCTACGAC |
| actin | CACCCGCGAGTACAACCTTC | CCCATACCCACCATCACACC |

**Supplementary Table S2.** pIL10 plasmid fragment sequences

| Fragment sequence | | | | | |
| --- | --- | --- | --- | --- | --- |
| atgcctggc | tcagcactgc | tatgttgcct | gctcttactg | gctggagtga | agaccagcaa |
| aggccattcc | atccggggtg | acaataactg | cacccacttc | ccagtcagcc | agacccacat |
| gctccgagag | ctgagggctg | ccttcagtca | agtgaagact | ttctttcaaa | agaaggacca |
| gctggacaac | atactgctga | cagattcctt | actgcaggac | tttaagggtt | acttgggttg |
| ccaagccttg | tcagaaatga | tcaagtttta | cctggtagaa | gtgatgcccc | aggcagagaa |
| ccatggccca | gaaatcaagg | agcatttgaa | ttccctggga | gagaagctga | agaccctctg |
| gatacagctg | cgacgctgtc | atcgatttct | cccctgtgag | aataaaagca | aggcagtgga |
| gcaggtgaag | aatgatttta | ataagctcca | agacaaaggt | gtctacaagg | ccatgaatga |
| gtttgacatc | ttcatcaact | gcatagaagc | ctacgtgaca | ctcaaaatga | aaaattga |

# Supplementary Figures


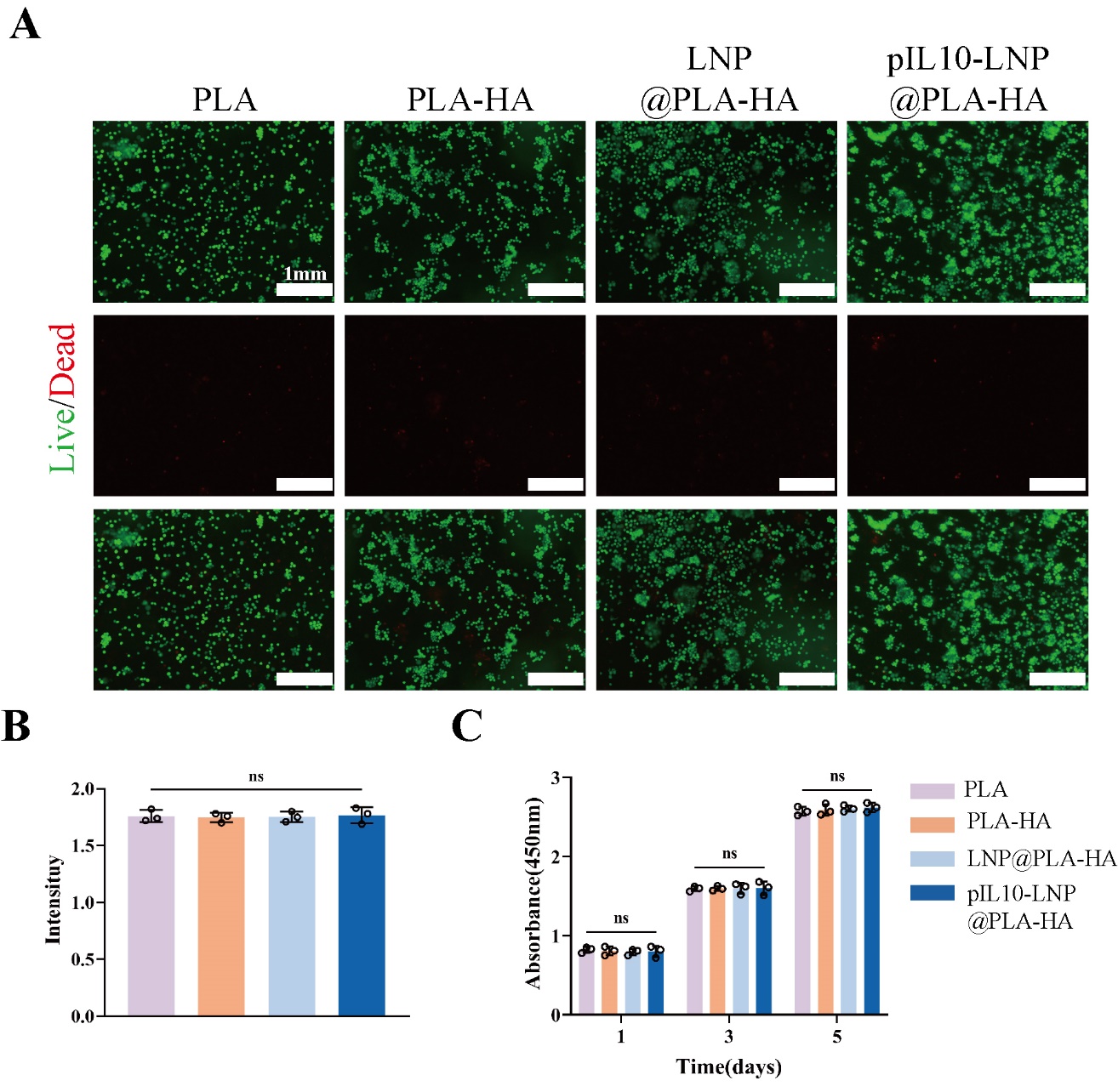


**Supplementary Figure 1 |** Cytocompatibility of different electrospun scaffolds. **(A)** Fluorograms of live-dead cell staining after 3-day co-culture of electrospun scaffolds and BMMs; **(B)** Fluorescence semi-quantitative analysis of live cells; **(C)** CCK-8 assay for proliferation of BMMs on different electrospun scaffolds after one, three and five days of co-culture. **p*<0.05, ***p*<0.01, ****p*<0.001 in one-way ANOVA and Tukey's post hoc test.


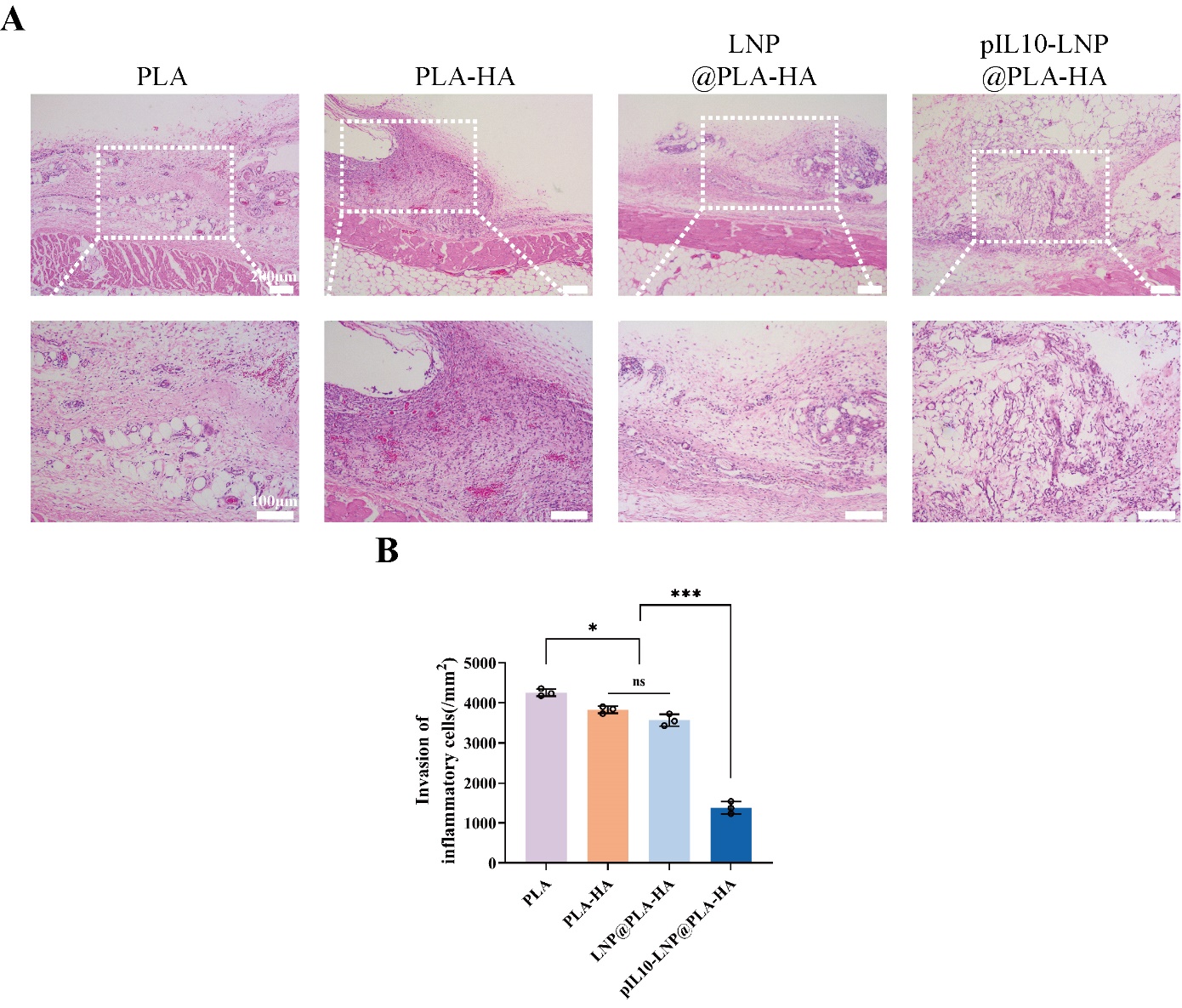


**Supplementary Figure 2 |** Compatibility assessment of electrospun scaffolds in animals in each group. **(A)** H&E staining two weeks after subcutaneous implantation of scaffolds in rats. **(B)** Quantitative analysis of inflammatory cells on electrospun scaffolds in each group. **p*<0.05, ***p*<0.01, ****p*<0.001 in one-way ANOVA and Tukey's post hoc test.


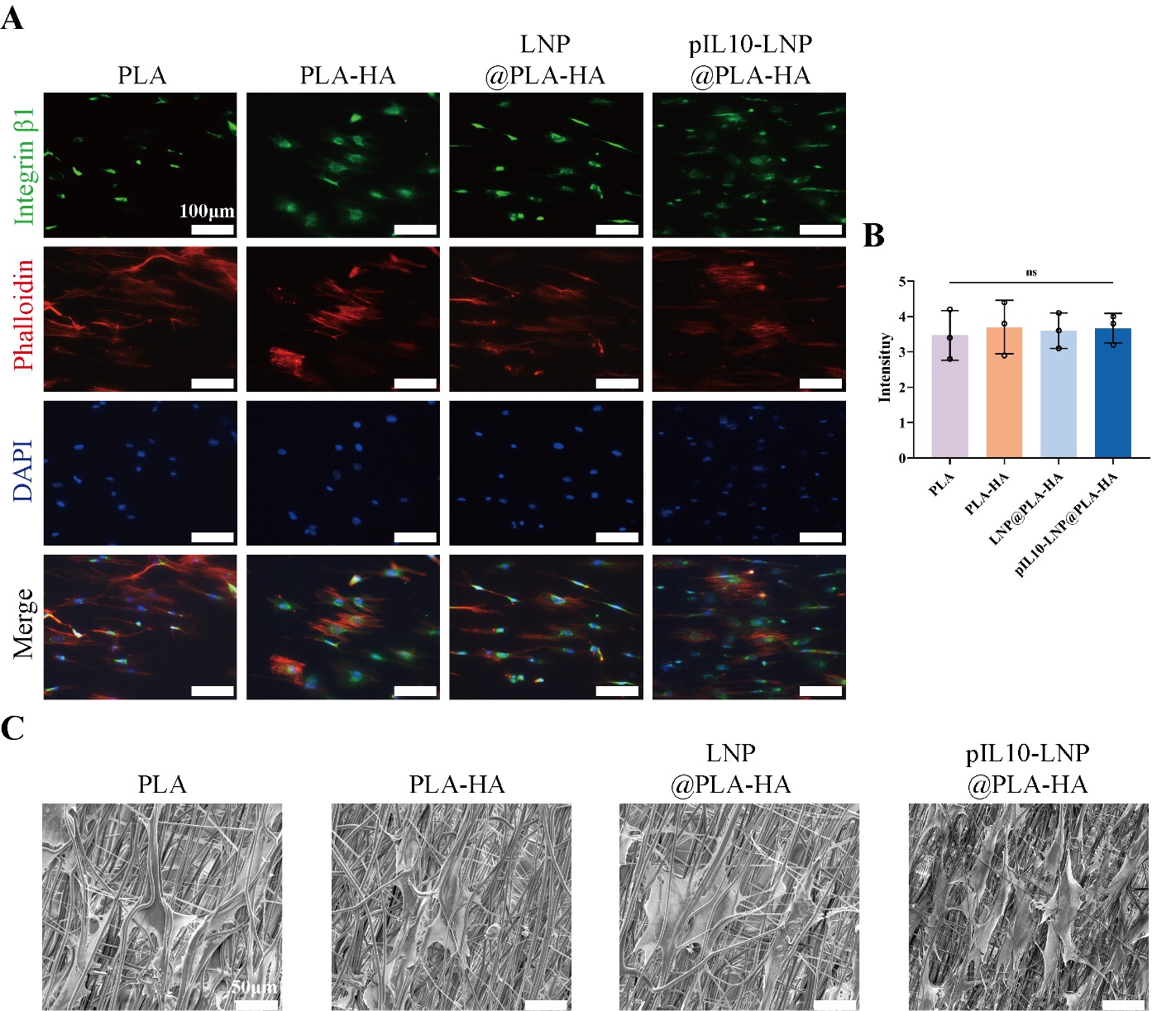


**Supplementary Figure 3 |** Cell adhesion on different electrospun scaffolds. **(A)** Images of IF staining of integrin β1 one day after implantation of NSCs in each group; **(B)** Fluorescence semi-quantitative analysis of integrin β1; **(C)** SEM images of NSCs on electrospun scaffolds. **p*<0.05, ***p*<0.01, ****p*<0.001 in one-way ANOVA and Tukey's post hoc test.


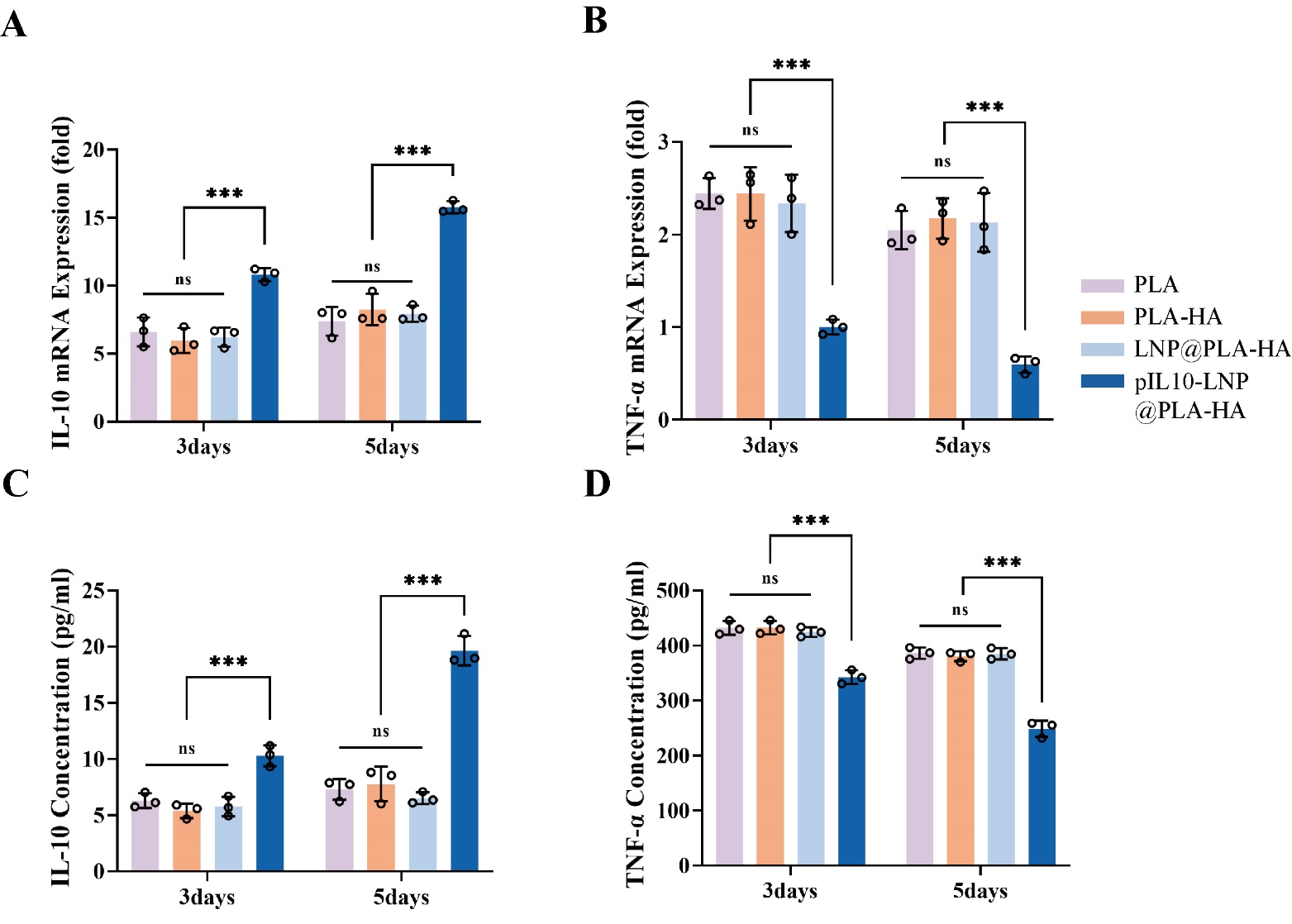


**Supplementary Figure 4 |** The impact of electrospun scaffolds on modulating immune function in BMMs. **(A-B)** The expression levels of the pro-inflammatory gene TNF-α and the anti-inflammatory gene IL-10 in BMMs; **(C-D)** The levels of the pro-inflammatory factor TNF-α and the anti-inflammatory factor IL-10 in BMMs. **p*<0.05, ***p*<0.01, ****p*<0.001 in two-way ANOVA and Tukey's post hoc test.


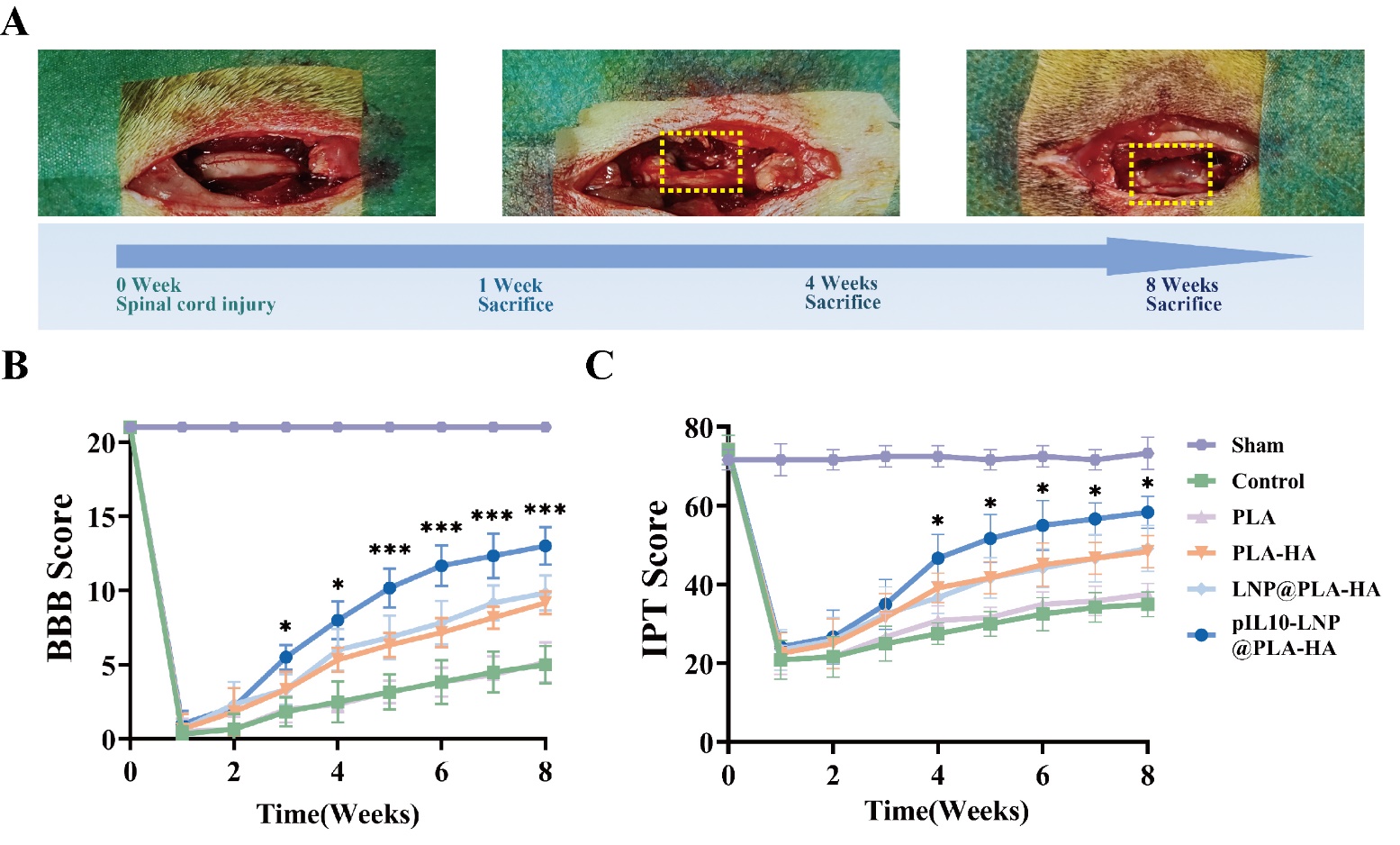


**Supplementary Figure 5 |** Timeline of animal experiments and motor function scores. **(A)** Surgical procedures of T9 hemi-transverse SCI model for scaffold implantation and time points of sampling in each group; **(B)** BBB scores of lower limbs of rats postoperatively; **(C)** IPT scores of rats. **p*<0.05, ***p*<0.01, ****p*<0.001 in one-way ANOVA and Tukey's post hoc test.


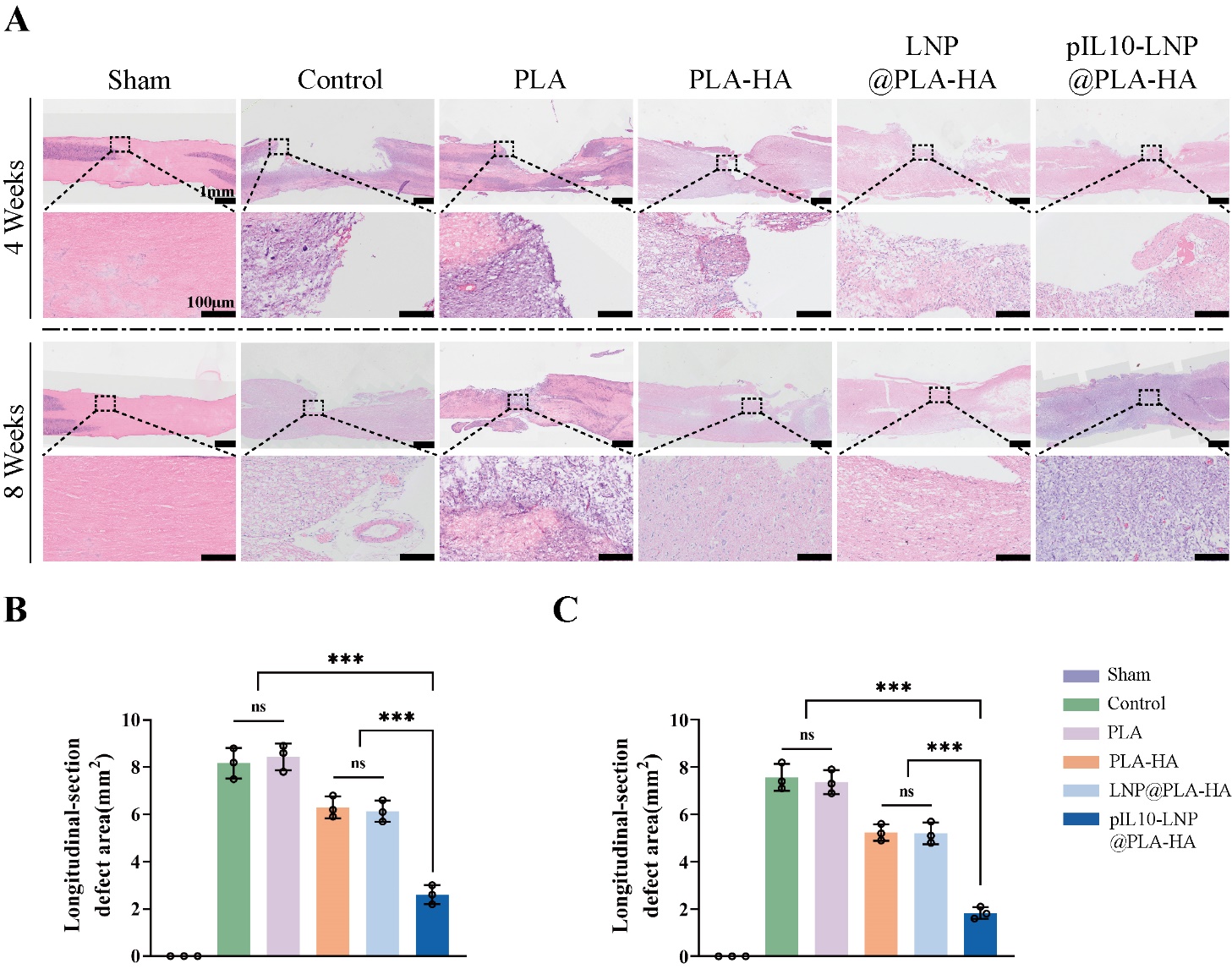


**Supplementary Figure 6 |** Pathological examination of T9 hemi-transverse SCI model for scaffold implantation in each group. **(A)** H&E staining four and eight weeks postoperatively in each group; **(B)** **(C)** Statistical analysis of spinal cord cavity area. **p*<0.05, ***p*<0.01, ****p*<0.001 in one-way ANOVA and Tukey's post hoc test.
