# Supplementary figures and images for: Genetically engineered electrospinning contributes to spinal cord injury repair by regulating the immune microenvironment
Source: Front Bioeng Biotechnol. 2024 Jun 12;12:1415527. doi: 10.3389/fbioe.2024.1415527 (PMC11199540; doi:10.3389/fbioe.2024.1415527)

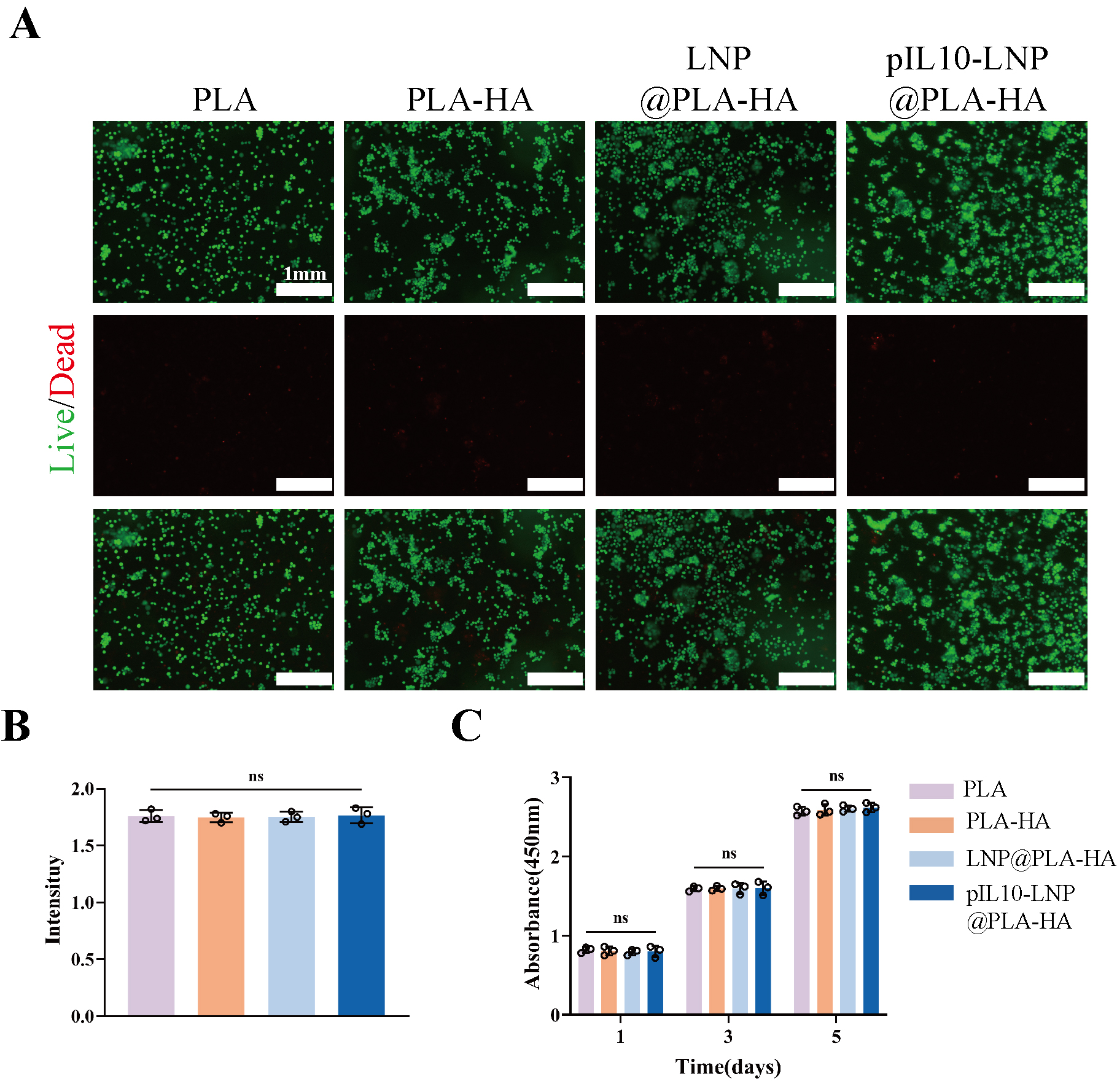

Supplement: Supplementary file 1 [file Presentation1.zip › Supplementary_Material/Fig S1.jpg]

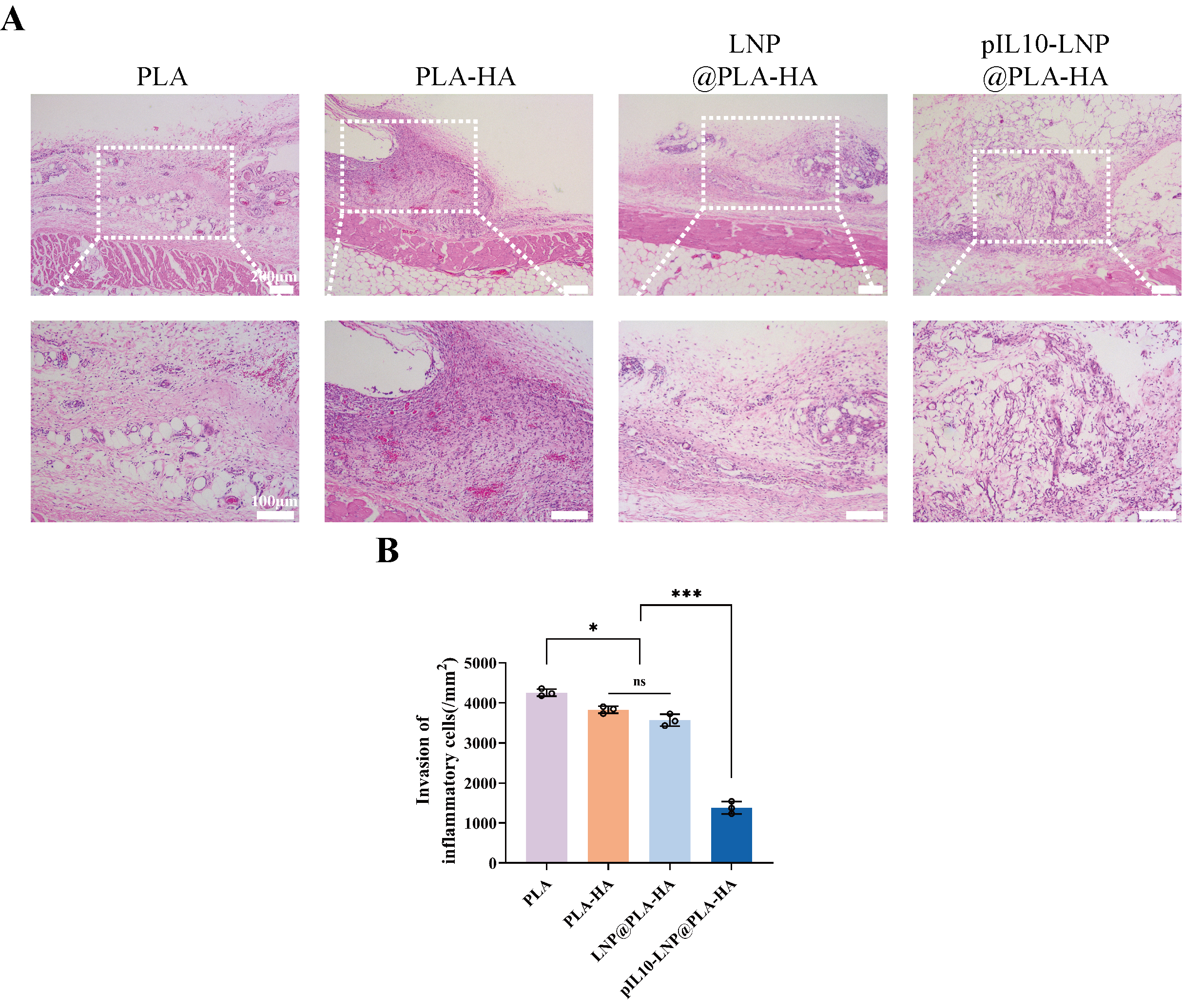

Supplement: Supplementary file 1 [file Presentation1.zip › Supplementary_Material/Fig S2.jpg]

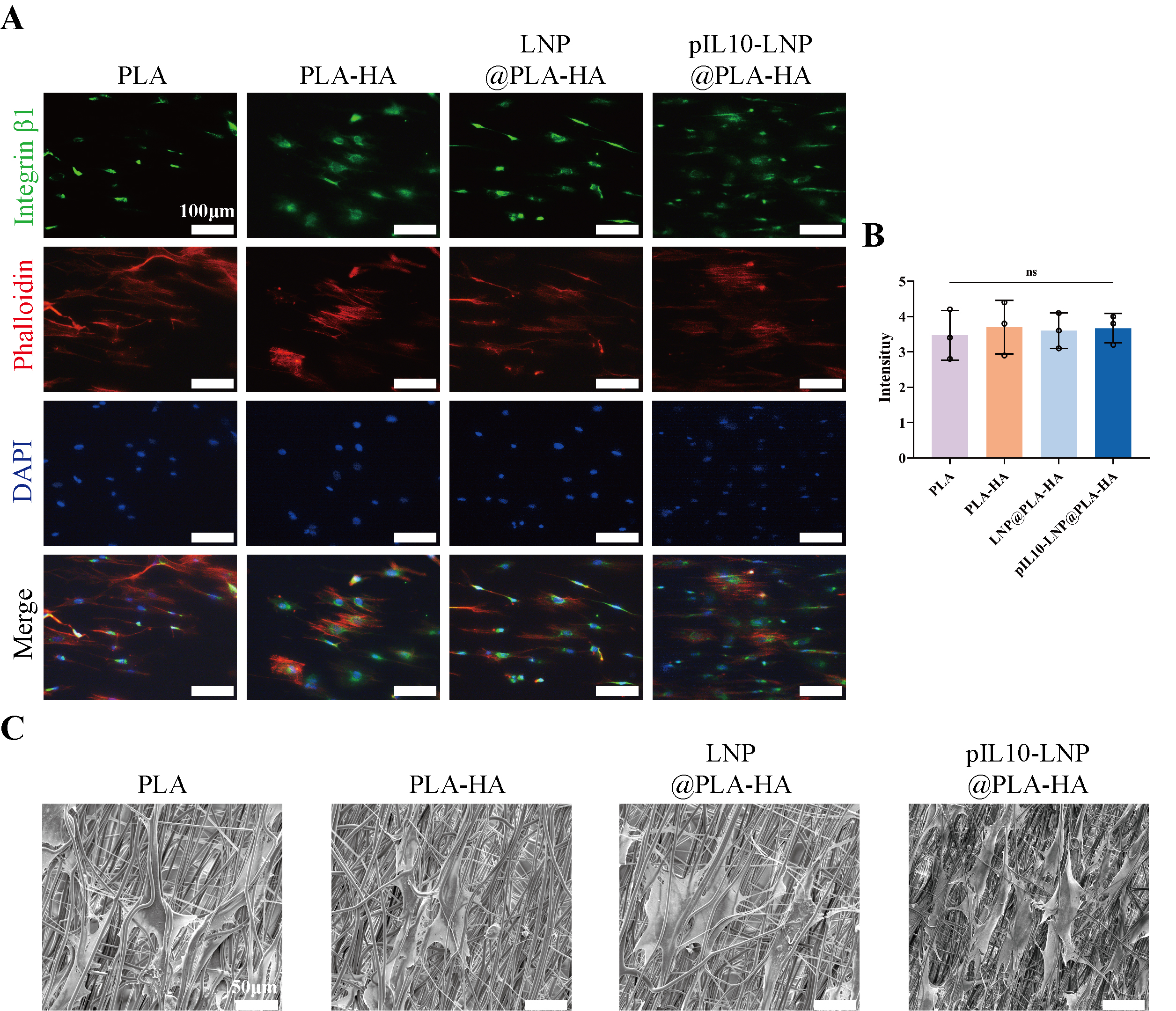

Supplement: Supplementary file 1 [file Presentation1.zip › Supplementary_Material/Fig S3.jpg]

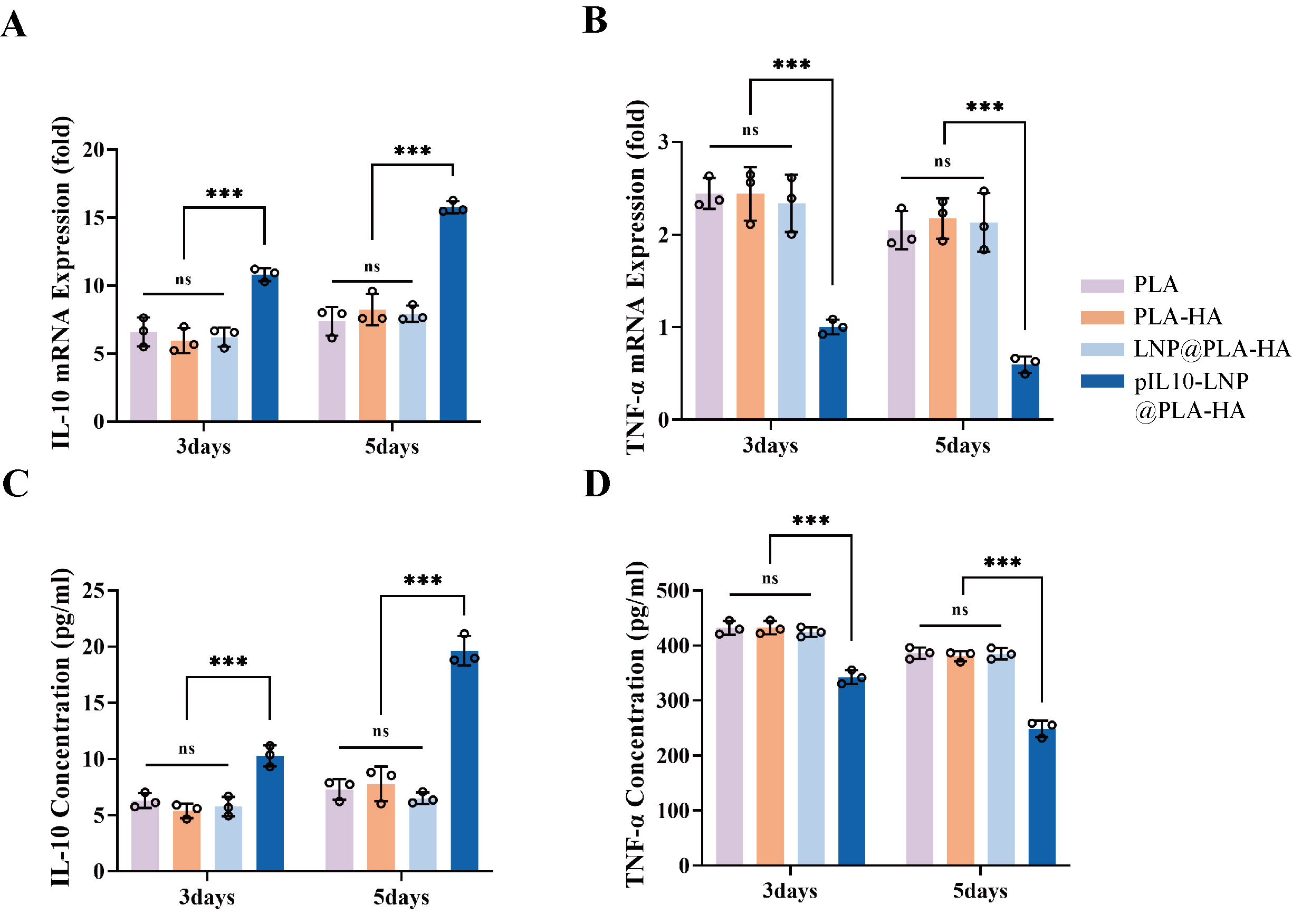

Supplement: Supplementary file 1 [file Presentation1.zip › Supplementary_Material/Fig S4.jpg]

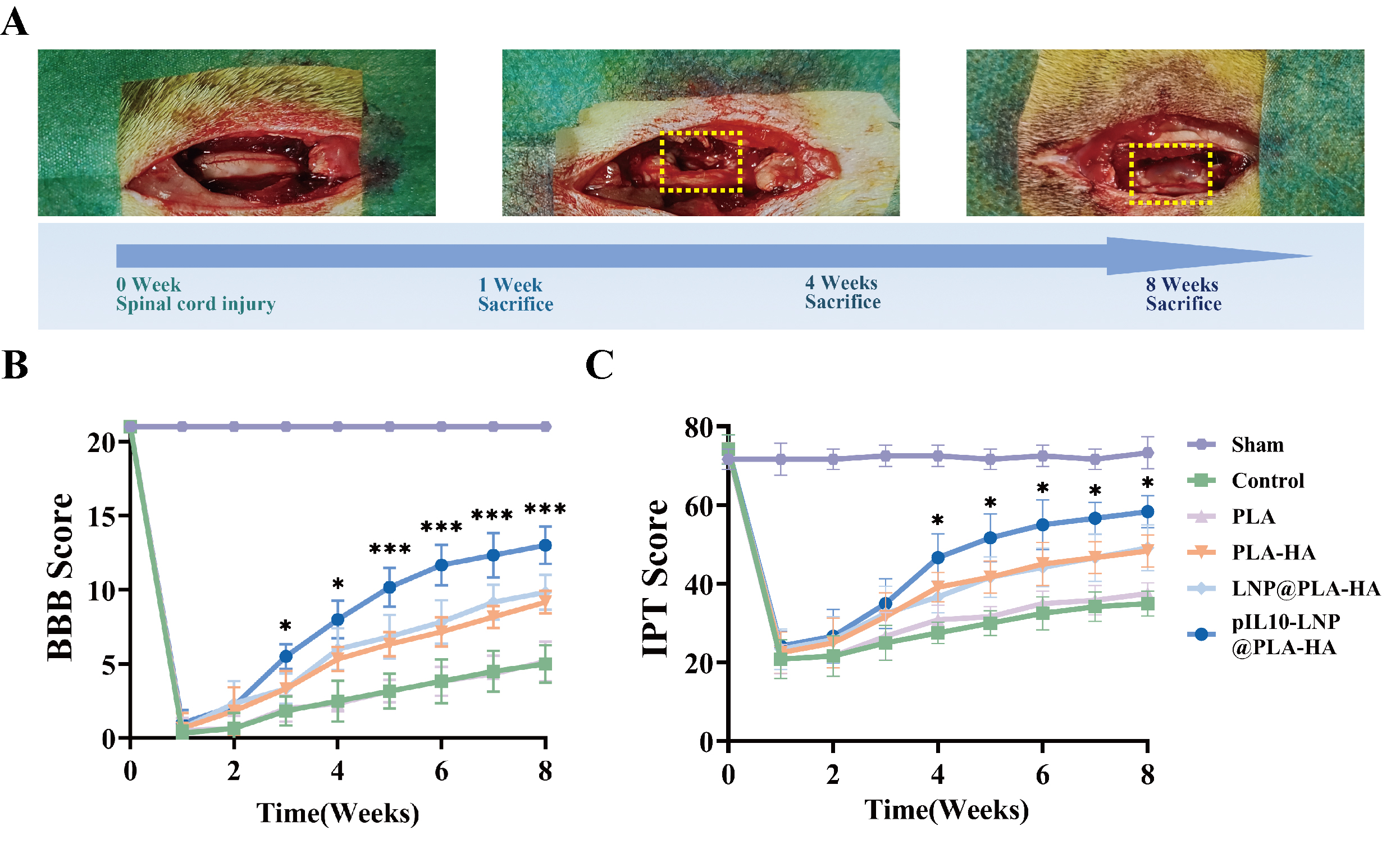

Supplement: Supplementary file 1 [file Presentation1.zip › Supplementary_Material/Fig S5.jpg]

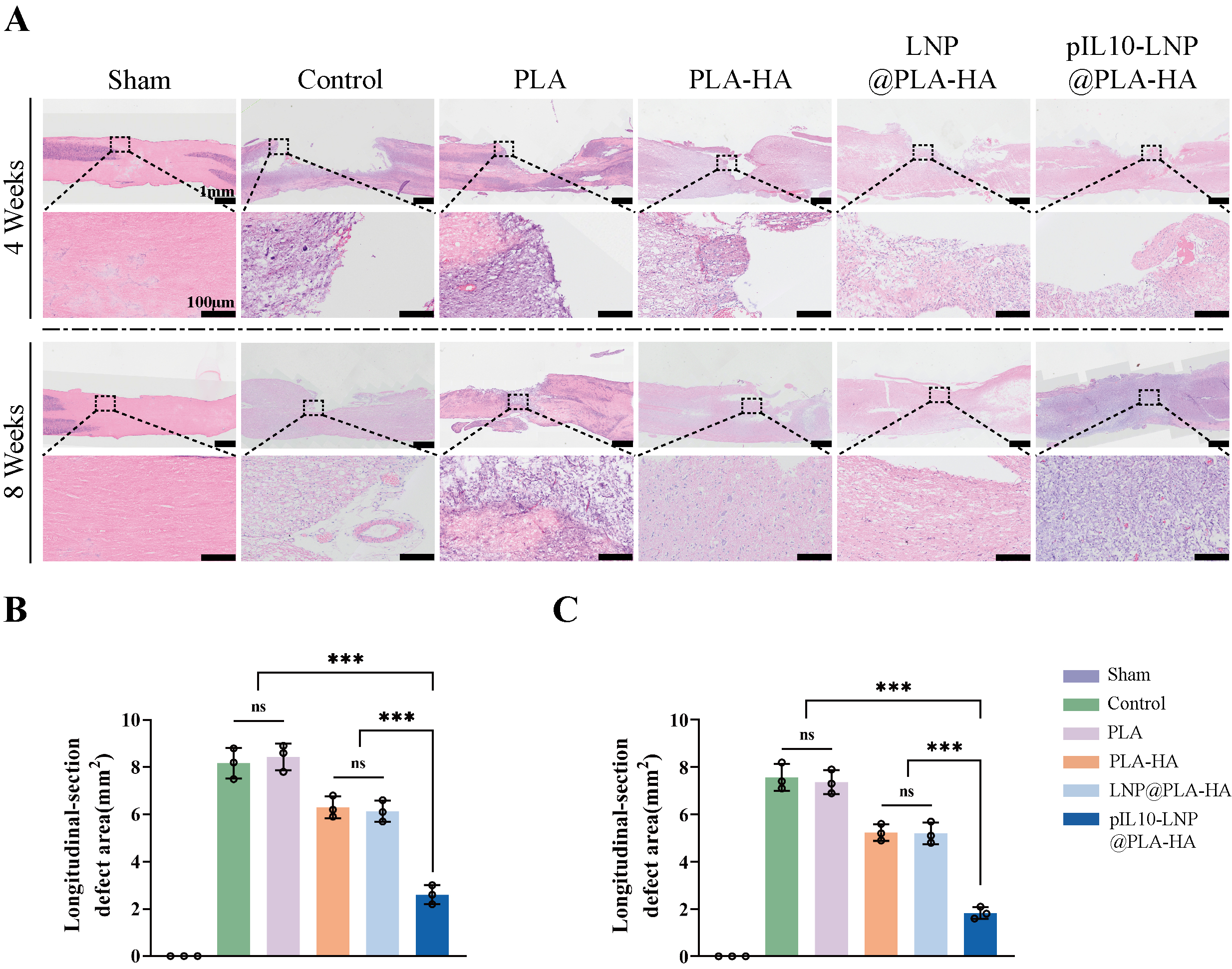

Supplement: Supplementary file 1 [file Presentation1.zip › Supplementary_Material/Fig S6.jpg]
